# Genetic analysis of resistance to stripe rust in durum wheat (*Triticum turgidum* L. var. *durum*)

**DOI:** 10.1371/journal.pone.0203283

**Published:** 2018-09-19

**Authors:** Xue Lin, Amidou N’Diaye, Sean Walkowiak, Kirby T. Nilsen, Aron T. Cory, Jemanesh Haile, Hadley R. Kutcher, Karim Ammar, Alexander Loladze, Julio Huerta-Espino, John M. Clarke, Yuefeng Ruan, Ron Knox, Pierre Fobert, Andrew G. Sharpe, Curtis J. Pozniak

**Affiliations:** 1 Department of Plant Sciences and Crop Development Centre, University of Saskatchewan, Saskatoon, SK, Canada; 2 International Maize and Wheat Improvement Center (CIMMYT), Mexico D.F., Mexico; 3 INIFAP, Campo Experimental Valle de México, Chapingo, Edo. de México, México; 4 Swift Current Research and Development Centre, Agriculture and Agri-Food Canada, Swift Current, SK, Canada; 5 National Research Council, Saskatoon, SK, Canada; 6 Global Institute for Food Security, University of Saskatchewan, Saskatoon, SK, Canada; Institute of Genetics and Developmental Biology Chinese Academy of Sciences, CHINA

## Abstract

Stripe rust, caused by the fungal pathogen *Puccinia striiformis* Westend. f. sp. *tritici* Eriks, is an important disease of bread wheat (*Triticum aestivum* L.) worldwide and there is an indication that it may also become a serious disease of durum wheat (*T*. *turgidum* L. var. *durum*). Therefore, we investigated the genetic architecture underlying resistance to stripe rust in adapted durum wheat germplasm. Wheat infection assays were conducted under controlled conditions in Canada and under field conditions in Mexico. Disease assessments were performed on a population of 155 doubled haploid (DH) lines derived from the cross of Kofa (susceptible) and W9262-260D3 (moderately resistant) and on a breeding panel that consisted of 92 diverse cultivars and breeding lines. Both populations were genotyped using the 90K single-nucleotide polymorphism (SNP) iSelect assay. In the DH population, QTL for stripe rust resistance were identified on chromosome 7B (LOD 6.87–11.47) and chromosome 5B (LOD 3.88–9.17). The QTL for stripe rust resistance on chromosome 7B was supported in the breeding panel. Both QTL were anchored to the genome sequence of wild emmer wheat, which identified gene candidates involved in disease resistance. Exome capture sequencing identified variation in the candidate genes between Kofa and W9262-260D3. These genetic insights will be useful in durum breeding to enhance resistance to stripe rust.

## Introduction

Durum wheat (*Triticum turgidum* L. var. *durum*) is an important food crop in regions with relatively dry climates. Worldwide, 21 countries produce durum wheat across an average area of approximately 18 million hectares, with an annual production of approximately 35 million tonnes (Mt) [1]. Canada is the second largest producer of durum wheat in the world, with Saskatchewan (SK), Alberta (AB) and Manitoba (MB) contributing 84%, 14% and 2% to national production, respectively [2].

Wheat stripe or yellow rust is caused by the fungus *Puccinia striiformis* Westend. f. sp. *tritici* Eriks (*Pst*). The pathogen is widespread in nearly all wheat growing areas on all continents and is estimated to cause 5.47 Mt of wheat losses annually, valued at approximately 979 million US dollars [3]. Regional production that is exposed to stripe rust persistently ranges from 73.9 to 89.0% in Europe (including Turkey), Latin America and the Caribbean, and sub-Saharan Africa [3]. In terms of global production, exposures are persistently greatest in Europe (including Turkey) (20.5%) and in the Pacific/Asia (4.5%), including China, the world’s largest producer of wheat [3, 4]. Trends also indicate increased losses from stripe rust since 2000, especially in North America [3]. Historically, stripe rust was not considered an economically important disease in Canada; however, stripe rust has appeared more frequently in western Canada since 2000 and during the recent stripe rust epidemics in 2010 and 2011, most Canadian commercial common wheat cultivars were infected [5, 6]. Most of the commercially grown durum wheat germplasm are resistant to stripe rust, but there are indications of increased incidence of the disease due to the appearance of new races with increased virulence on durum, such as those identified in Mexico in 2014 and 2016 [7].

The primary host of *Pst* is wheat, with barberry (*Berberis* spp.) and Oregon grape (*Mahonia aquifolium*) serving as alternate hosts [8, 9]. However, eradication of barberry, which also serves as an alternate host for *P*. *graminis* f. sp. *tritici* (stem rust), has limited the lifecycle of *Pst* to wheat in most parts of North America [10, 11]. *Pst* is an obligate biotrophic parasite that absorb nutrients from living tissues [12]. After approximately two weeks, the fungus produces yellow uredia on the surface of the leaves, which appear in linear arrangements. The urediniospores within the uredia can be dispersed, by wind, thousands of kilometers from the initial infection site, thereby allowing the pathogen to colonize new wheat plants. Because of the short time required for the fungus to generate urediniospores, multiple generations of *Pst* can develop in a single growing season [4, 12]. *Pst* is adapted to cool environmental conditions (7–20°C) and may survive under mild winter conditions in Canada by reproducing asexually. However, temperatures are too severe in most northern locations for *Pst* to survive and the disease cycle usually ends with the wheat harvest [3, 4]. In western Canada, the source of inoculum in the spring is influenced by epidemics in the south and the Pacific North West; however, the geographic range of stripe rust is expanding as a result of changes in climate and pathogen adaptation, thus highlighting the importance of studying this pathogen in both northern and southern climates of North America [13, 14].

Sowing wheat cultivars that express adequate levels of resistance to stripe rust is the most effective strategy to control the disease [15, 16]. In general, there are two types of resistance: all-stage or seedling resistance and adult-plant resistance (APR). All-stage resistance is usually expressed in all plant growth stages and is generally only effective against specific races of the pathogen (race-specific). Due to the rapid evolution of the pathogen population, all-stage resistance genes deployed in a monogenic state are prone to rapid breakdown [17]. In contrast, APR is only expressed from approximately stem elongation to early head emergence, with maximum expression occurring during the boot stage [18]. Adult-plant resistance can also be effective against multiple races of *Pst* in high temperature environments. To date, there are only four well-characterized APR resistance genes that are effectively and knowingly used in field breeding programs. These include *Lr34*/*Yr18* (on chromosome 7DS), *Lr46*/*Yr29* (on chromosome 1BL), *Lr67*/*Yr46* (near the centromere of chromosome 4DL) and *Sr2*/*Yr30* (on chromosome 3BS) [19, 20]. *Lr34/Yr18* and *Lr67/Yr46* are not readily available to tetraploid durum wheat breeders because of their location on the D-genome of hexaploid wheat. Even though these genes are race-non-specific, wise gene stewardship would be that new genes, those not already widely used in bread wheat, should be the focus in durum wheat breeding. Thus, efforts are needed to identify new sources of resistance for durum wheat, including genes that can be combined or pyramided for durable resistance.

Combining multiple resistance genes into breeding lines can be difficult if based exclusively on phenotypic selection, especially when all-stage resistance genes are involved. As an alternative to phenotypic selection alone, molecular markers that are tightly linked to resistance genes are used for stacking resistance genes [21]. Although numerous studies have investigated and developed genetic markers for stripe rust resistance, research has primarily focused on hexaploid wheat and much less is known about the genetic basis of stripe rust resistance in durum wheat or from other tetraploid sources. Among the 78 officially named resistance genes to date [22], roughly seven were detected in durum wheat [22–24].

The aim of this study was to identify additional stripe rust resistance genes in modern durum wheat germplasm that could be used in breeding programs to develop more durable resistance. The 90K SNP iSelect assay made it possible to genotype a large number of accessions and simultaneously investigate the genetic architecture of stripe rust resistance in durum wheat. We performed linkage analysis on a bi-parental doubled haploid (DH) mapping population from the cross of Kofa (susceptible) and W9262-260D3 (moderately resistant), which was evaluated for seedling resistance against two *Pst* isolates. QTL from the DH population were also tested in a breeding panel consisting of 92 diverse durum cultivars and breeding lines.

## Materials and methods

### Populations

Two populations were used in the present study; the first was a bi-parental population consisting of 155 DH lines, derived from the cross between the susceptible parent Kofa and the moderately resistant parent W9262-260D3 (Kyle*2/Biodur) [25]. The second population consisted of a breeding panel of 92 elite cultivars and breeding lines collected from 13 countries, representative of the major durum wheat breeding programs of the world (S1 Table). In addition, three stripe rust susceptible checks (Avocet, Brigade and DT749) and two resistant checks (DT546 and Lillian) were included in the panel.

### Collection of *Pst* races

Two isolates (W009 and W015) and a field collection (FC) of stripe rust were provided by the Cereal & Flax Pathology Laboratory at the Crop Development Centre, University of Saskatchewan. W009 was isolated in Richardson, SK (50° 24’N, 104° 29’W) in 2011 and W015 was isolated in Lethbridge, AB (49° 43’N, 112° 48’ W) in 2010 [13]. These isolates were propagated to obtain inoculum by infecting susceptible wheat plants (cv. ‘Avocet S’) with urediniospores that were obtained from a single pustule. Brar and Kutcher [13] reported that W009 and W015 are genetically uniform isolates with different avirulence / virulence formulae. Isolate W009 is race C-PST-2, which is the second most common race among 59 isolates collected from 2005 to 2013 in western Canada. Isolate W015 is race C-PST-30, and was determined previously to have the widest spectrum of virulence among western Canadian isolates when evaluated on the Avocet differential set [13].

The FC for the seedling assays was collected from naturally infected susceptible spring wheat lines in Lethbridge, Alberta in 2011, and the composition is unknown. Stripe rust urediniospores were collected from multiple infected wheat leaves, combined, and stored in a freezer at -80°C. The FC was also propagated on the susceptible cultivar ‘Avocet S’ to obtain additional inoculum.

### Stripe rust resistance evaluation

Evaluation of seedling stripe rust resistance was performed in an environmentally controlled growth chamber with a diurnal temperature cycle of 18°C in darkness and 22°C in light, with a photoperiod of 8 h of darkness and 16 h of light. Seedlings of both populations were inoculated at the two-leaf stage (approximately 10 days after planting). Inoculations were made using urediniospores suspended in mineral oil (Bayol®, Esso Canada, Toronto, ON) at a concentration of 0.01 g of urediniospores per 900 μl mineral oil. Inoculated seedlings were left to dry and transferred to a high humidity growth chamber (10°C) and kept in darkness for 24 h. Seedlings were then moved to a growth chamber with a temperature of 10°C in darkness and 15°C in light, with a photoperiod of 8 h of darkness and 16 h of light. The DH population was inoculated with spores of W009 and W015 as independent experiments, while the breeding panel was inoculated with W009, W015, and FC, as independent experiments. The DH population and the breeding panel were arranged in an alpha-lattice design with three replications and four seedlings per replication (S2 Table). Ten to eighteen days post-inoculation, the second leaf was evaluated for disease based on the 0 (resistant) to 9 (susceptible) scale rating of infection type (IT) [12, 18]. The IT was scored twice approximately two days apart, and scores were analyzed independently.

The DH population was also evaluated for stripe rust resistance in replicated field trials in Mexico in 2014. Similarly, the breeding panel was evaluated in Mexico over two consecutive years, 2013 and 2014. The experiments were conducted in Toluca (19°17’N, 99°39’W, 2,680 m above sea level, sandy clay loam soil). A randomized complete block design (RCBD) with three replications was employed in each field test (S3 Table). The experimental lines were planted in single row plots (1.5 m) with 0.5 m space between rows, with approximately 30 plants per plot. Susceptible spreader rows were planted around the experimental plots to facilitate the spread of the disease. Both spreader rows and experimental plots were artificially inoculated with a mixture of stripe rust races with the widest virulence spectrum in Mexico. Disease severity (DS) in the field trials was evaluated for the flag leaf based on the modified Cobb scale, on a per plot basis [26]. The first disease evaluation in 2014 was performed when the flag leaves of susceptible checks reached 40% severity. For both 2013 and 2014, subsequent ratings were performed every five days with the final rating performed when the susceptible checks reached 100% severity, at approximately Zadoks growth stage (GS) 55 [27]. The area under the disease progress curve (AUDPC) for the field data collected in Mexico was used to obtain an estimate of disease accumulation on each plot. The AUDPC was calculated using the following formula; where, *n* is the total number of ratings, Y_i_ is the stripe rust severity for the ^*i*^th rating and *T_i_* is the day of the ^*i*^th rating [28].

$$AUDPC=\sum_{i=1}^{n}\left[\frac{Y_{i}+Y_{i-1}}{2}]\;(T_{i}-T_{i-1}\right)$$

The disease evaluation data was analyzed using PROC MIXED in SAS V9.3 with durum accessions as a fixed effect, while replications (Rep), blocks and interacting factors were considered random. The least square means (LSMeans) for the stripe rust disease ratings were calculated using LSMEANS in SAS V9.3 for both growth chamber and field experiments [29]. The deviations of observed and expected frequencies of resistant individuals in the DH population were tested using the Pearson’s chi-squared test.

### DNA extraction, genotyping, and construction of the genetic map for the DH population

Genomic DNA was extracted from leaves of one-week-old seedlings using the cetyltrimethylammonium bromide (CTAB) protocol [30]. Standard gel electrophoresis, using a 1.5% (w/v) agarose gel with known size standards, was used to evaluate the quality and integrity of the DNA samples. DNA was quantified by the PicoGreen fluorometric assay [31]. Each sample was genotyped using 500 ng of DNA in 10μl as input for the Wheat 90K iSelect SNP assay [32]. Genotypic data was analyzed by the genotyping module of Illumina GenomeStudio data analysis software GSGT V1.9.4 (Illumina, San Diego, CA). For the DH population, in addition to the 10,641 SNP markers from the 90K SNP Infinium iSelect assay [32], 109 SSR and 125 DArT markers [25] were analyzed. The polymorphism information content (PIC) of both SNP and SSR markers were determined by PowerMarker V3.25 [33]. A genetic map of the DH population was constructed using both MSTMap [34] and MapDisto [35], as described previously [36]. For the breeding panel, conservative filtering (S4 Table) was applied to filter out non-informative SNPs. There were 244 simple sequence repeat (SSR) markers that were also used in the analysis of the breeding population [37].

### QTL analysis

Composite interval mapping (CIM) was performed by Qgene V4.3.10 [38], using the genetic map of the DH population. We used a stepwise cofactor selection method, where the ‘maximum number of cofactors’, ‘F-to-add’, and ‘F-to-drop’ thresholds were set as ‘auto’. One thousand permutation tests were performed to estimate the critical logarithm of the odd (LOD) threshold. Significant QTL were illustrated with diagonally hatched bars using MapChart V2.2 [39]. The additive and epistatic effects of QTL were investigated using SAS V9.3.

### Confirmation of QTL from the DH population in the breeding panel

Using data from the breeding panel, the linkage disequilibrium (LD) between pair-wise markers was calculated among SNPs with known genetic positions according to the SNP-based consensus map of durum wheat [40]. Using TASSEL V3.0, LD was measured using the squared correlation between loci (*r*^*2*^) and was plotted against genetic distance between adjacent markers. The LD decay against genetic distance was simulated in a nonlinear regression model [41]. The critical *r*^*2*^ value referred to the 95% quantile of *r*^*2*^ values between unlinked SNP markers (i.e. markers that were localized to different chromosomes).

The phylogenetic tree was constructed using allele frequency-based distances between accessions. Population structure within the breeding panel was estimated using principal component analysis (PCA) using TASSEL V3.0 [42], and Bayesian clustering analysis was performed using STRUCTURE V2.3.4 and Structure Harvester [43] to determine the coancestry coefficient (Q matrix). The Bayesian clustering analysis was applied to SSR markers with high PIC identified previously [37]. The Q matrix was used as a covariate in the general linear model (GLM) or mixed linear model (MLM). The kinship matrix (k matrix) was estimated using genetic data in TASSEL V3.0, and incorporated as a random effect in the MLM.

Association analyses were performed using three different models in TASSEL V3.0; these included a naïve model (GLM without any correction for population structure), GLM (with Q matrix as a correction for population structure) and MLM (with Q and P matrix as corrections for population structure) [44]. The model that best corrected for bias in population structure and kinship was chosen based on minimizing systematic inflation or deflation of *P*-values using the Quantile-Quantile (Q-Q) plot. The *P*-value was adjusted using a positive false discovery rate (pFDR) method. When the MLM was employed, an ‘FDR *Q*-value’ was estimated using the R package ‘fdrtool’ and *Q* ≤ 0.05 was used as threshold to determine significant associations [45, 46].

### Physical anchoring of QTL and exome sequencing

The genome sequence for wild emmer wheat (WEW) was used to determine the physical interval of the QTL. Markers were compared to the genome by GMAP v2014-12-29 [47] and filtered for matches with 95% sequence identity and 80% coverage. Genes within the intervals were extracted from the available gene annotations of WEW. For exome sequencing, DNA from Kofa and W9262-260D3 were enriched for coding regions using the wheat exome capture array according to the procedures outlined previously [48]. High-throughput sequencing was performed on the Illumina HiSeq2500 platform with 2 x 100 bp PE chemistry. Raw sequence reads were deposited in the NCBI Sequence Read Archive (Accession: SRP154228). Reads were processed in Trimmomatic v0.32 [49] and aligned to the genome of WEW using Novoalign v3.02.05. Duplicate read mappings and improper read pairs were removed using samtools v1.3.1 [50] and picard-tools [51]. Sequence variations were determined using freebayes v1.0.2-16-gd466de [52], with ploidy set to 4. Predictions on the effect of the variants on gene function were determined within high confidence gene models by SnpEff [53].

## Results

### Genetic mapping for stripe rust resistance in the DH population

**Fig 1:**
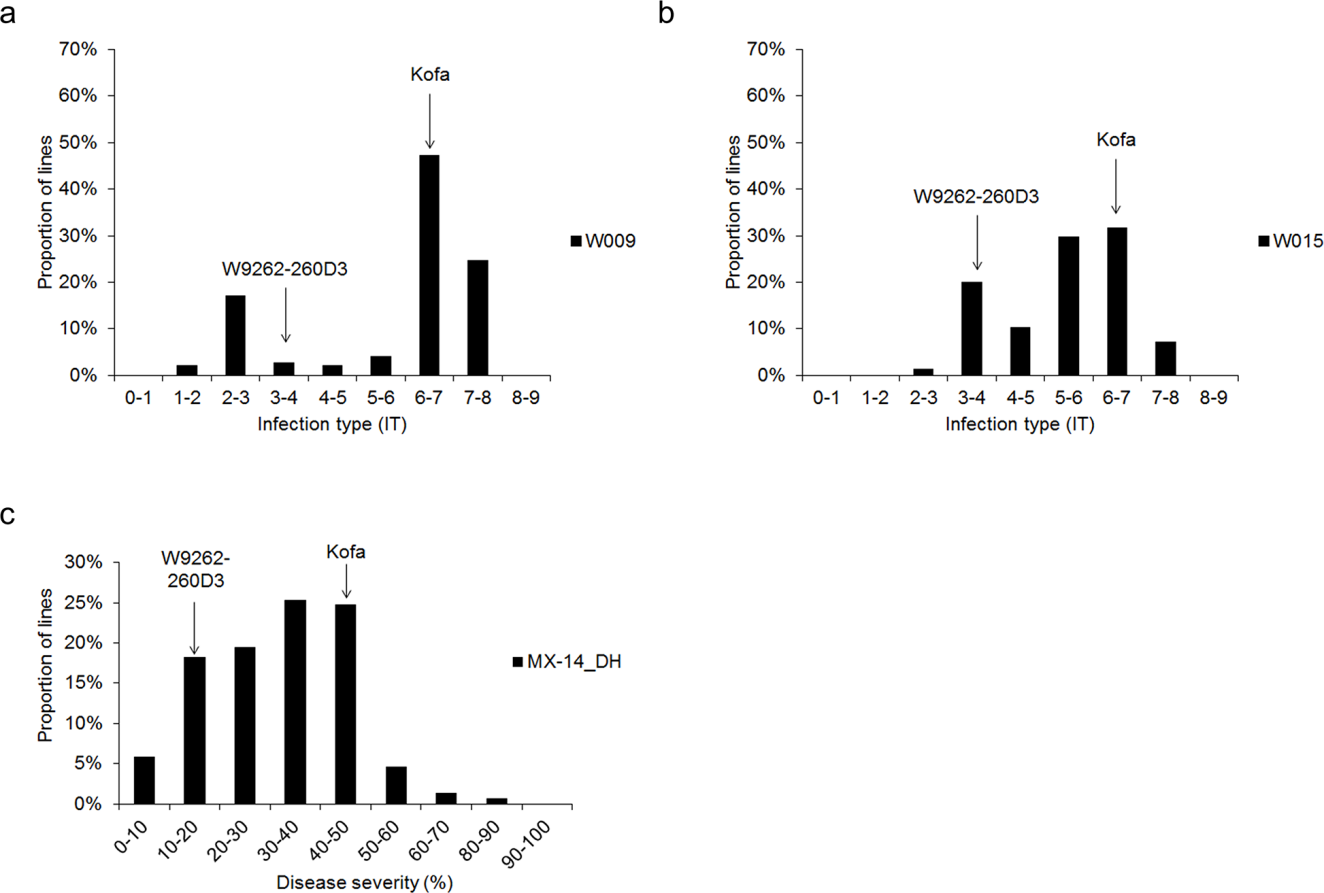
Frequency distributions for stripe rust disease for the DH population Kofa/W9262-260D3. Seedling disease reaction to (a) W009 and (b) W015. (c) Final scoring of adult plant disease severity collected on 27-Aug-2014 in Toluca, Mexico (MX-14_DH).

The two parents of the DH population had clear differences in their phenotypic response to stripe rust infection. In the seedling stage, the 1^st^ and 2^nd^ leaves of the moderately resistant parent W9262-260D3 had few uredia that were surrounded by chlorotic/necrotic areas, which were staggered on the leaf surface (IT 3–4). In contrast, both leaves of the susceptible parent Kofa were covered with many uredia, which showed no necrosis or chlorosis (IT 6–7). There was no observable difference in the number of uredia between isolates W009 and W015. The IT frequency distribution approximated a bimodal distribution in the DH population, with a range of 1–8 for isolate W009 and 2–8 for isolate W015 (Fig 1A and 1B). The frequency distribution of resistance to W009 had two peaks, with 17.1% of individuals with IT ranging from 2 to 3, and 71.9% of individuals with a more susceptible IT range of 6 to 8 (Fig 1A). The frequency distribution of resistance to W015 also had two peaks, with 20.0% of individuals with IT ratings that ranged from 3 to 4, and 61.3% of individuals with IT ratings that ranged from 5 to 7 (Fig 1B). Transgressive segregation for seedling resistance was also observed for the DH population. When inoculated with W009, 18.7% of the DH population had a higher level of resistance than W9262-260D3, while 23.2% were more susceptible than Kofa; similarly; for W015, 12.3% of the DH population were more resistant than W9262-260D3, while 35.5% were more susceptible than Kofa.

The distribution of stripe rust DS assessed in field experiments in Mexico in 2014 is shown in Fig 1C. The moderately resistant parent W9262-260D3 had some uredia on the surface of the flag leaf (DS of 10–20%), while susceptible parent Kofa had uredia on half of the flag leaf area (DS of 50%). DS of the DH population ranged from 0 to 90%; however, DS ranged from 10% to 50% for 87.7% of the population. Similar to the seedling reactions, the DH population also showed transgressive segregation for adult plant resistance to stripe rust (Fig 1). For adult plant resistance in Mexico in 2014, 5.8% of the DH population were more resistant than W9262-260D3, whereas 6.5% were more susceptible than Kofa.

**Fig 2:**
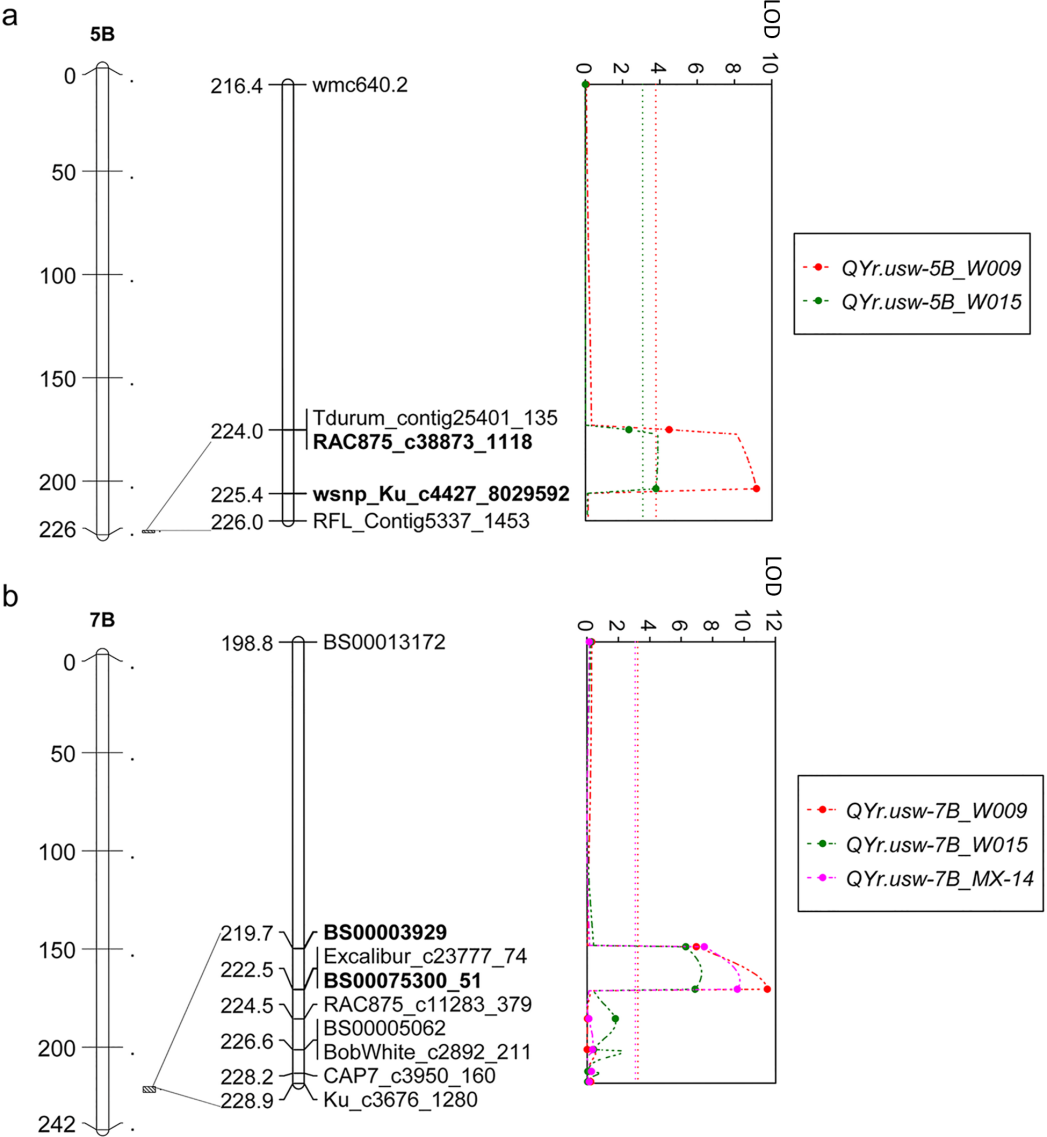
Map of stripe rust resistance QTL in the Kofa/W9262-260D3 DH population. The positions of significant QTL on chromosome 5B (a) and 7B (b) are illustrated by diagonally hatched bars next to the chromosome, which are expanded to show map detail. Flanking markers are in bold. The dotted lines indicate the QTL significance thresholds. The QTL are labelled and colored according to three independent experiments involving stripe rust infection: two growth cabinet experiments using single isolates W009 (orange) or W015 (green) and a third field experiment performed in Mexico in 2014 (MX-14, pink).

**Fig 3:**
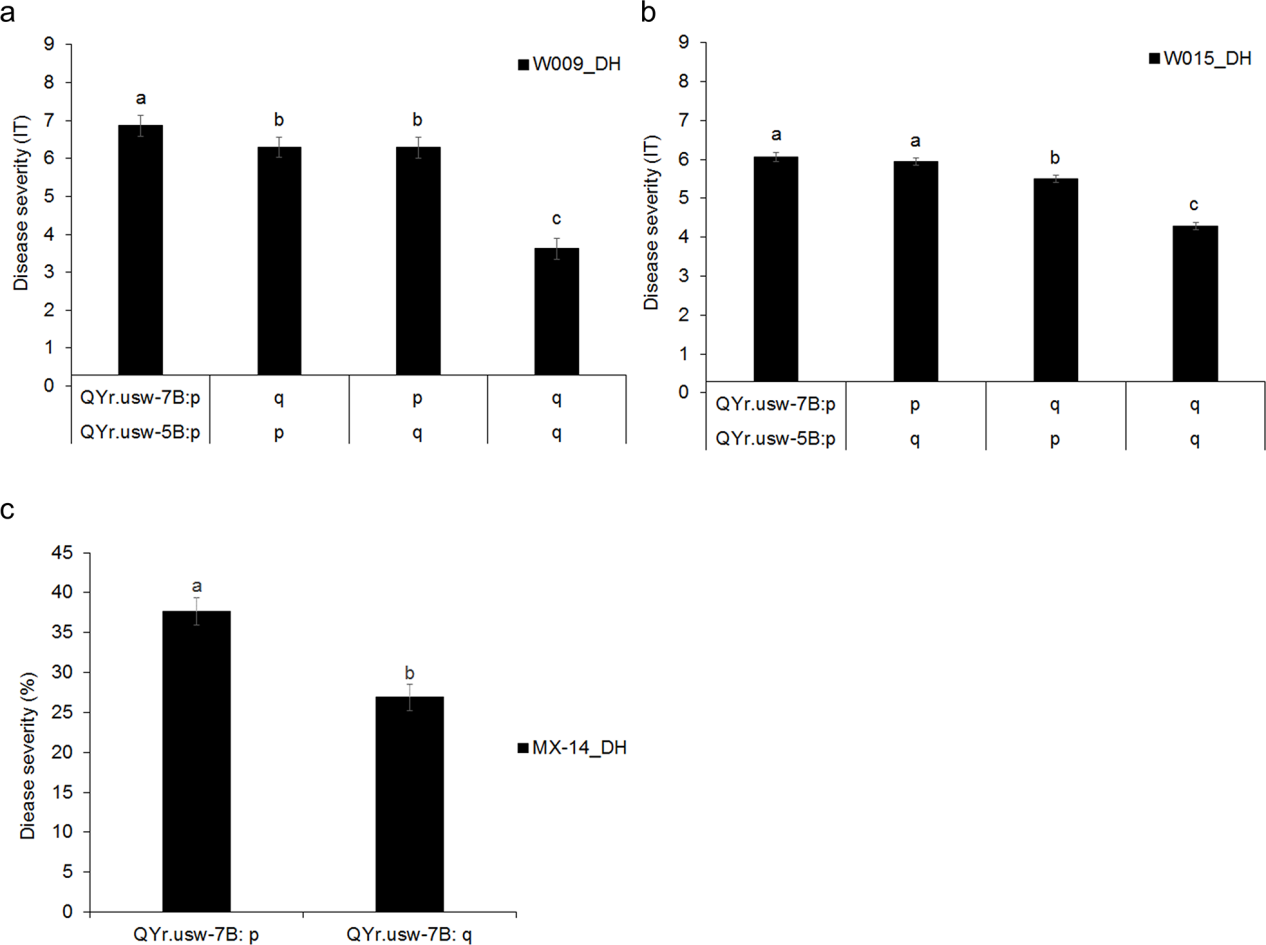
Mean stripe rust infection in the DH population Kofa/W9262-260D3 for QTL on chromosomes 7B and 5B. Seedling stripe rust reaction to (a) isolate W009 (W009_DH) and (b) W015 (W015_DH), and (c) adult plant resistance in Mexico in 2014 (MX-14_DH). The ‘p’ represents the susceptible allele from Kofa, while ‘q’ represents the resistant allele from W9262-260D3, as determined using the peak markers for *QYr*.*usw-5B* (*wsnp_Ku_c4427_8029592*) and *QYr*.*usw-7B* (*BS00075300_51*). There were 34, 37, 39, and 39 lines with the ‘pp’, ‘qp’, ‘pq’ and ‘qq’ haplotype for *QYr*.*usw-5B* and *QYr*.*usw-7B*, respectively (a and b). Likewise, there were 73 lines with the ‘p’ haplotype and 76 lines with the ‘q’ haplotype for *QYr*.*usw-7B* (c). Bars that do not share a letter differ significantly (Fisher’s LSD, *P < 0*.*05*).

In order to identify QTL associated with the resistance in the DH population, we first constructed a genetic map using genotypic data from the 90K iSelect SNP array. The genetic map spanned 2,639.7 cM with an average interval size of 0.64 markers/cM. Gaps larger than 10 cM were found on chromosomes 1A, 1B, 3A, 3B, 4A, 5A and 6A (S5 Table). Two QTL for seedling resistance to isolates W009 and W015 were then identified by CIM (permutation *P <* 0.05), *QYr*.*usw-5B* and *QYr*.*usw-7B* (Fig 2; Table 1; S1 Appendix). The moderately resistant parent W9262-260D3 carried the resistant alleles for both *QYr*.*usw-5B* and *QYr*.*usw-7B*. The QTL positions also coincided perfectly for both fungal isolates. *QYr*.*usw-7B* had the largest effect and was also identified in the field reaction to the Mexican races in Toluca in 2014 (Fig 2, Fig 3C and Table 1). *QYr*.*usw-7B* was flanked by markers *BS00003929* and *BS00075300_51* with an interval of 2.9 cM; the peak marker, *BS00075300_51*, was located at 222.5 cM on chromosome 7B. *QYr*.*usw-5B* was identified in seedling experiments, but was not detected in field trials against the Mexican races in 2014. *QYr*.*usw-5B* was flanked by markers *RAC875_c38873_1118* and *wsnp_Ku_c4427_8029592* with an interval of 1.3 cM; the peak marker, *wsnp_Ku_c4427_8029592*, was located at 225.3 cM on chromosome 5B. Based on seedling stripe rust resistance to W009 and W015, there was a significant epistatic interaction between *QYr*.*usw-5B* and *QYr*.*usw-7B* (*P <* 0.01). The two-QTL interaction explained 12.7% of seedling resistance to W009 and 17.1% for W015 (Fig 3A and 3B).

**Table 1:** QTL for stripe rust resistance to W009, W015 and Mexican races identified in the Kofa/W9262-260D3 DH population.

QTL	Chr.	Resistance interaction	Flanking markers	LOD	*R*^2^ (%)	Additive effect
*QYr*.*usw-5B*	5BL	Seedling—W009	*RAC875_c38873_1118—wsnp_Ku_c4427_8029592*	9.17	25.1	0.79
		Seedling—W015		3.88	11.0	0.35
*QYr*.*usw-7B*	7BL	Seedling—W009	*BS00003929—BS00075300_51*	11.47	30.4	0.90
		Seedling—W015		6.87	18.6	0.49
		Adult— Mexico ^a^		9.57	25.0	2.20

^a^ Data are from a field experiment performed in Mexico in 2014.

### Disease reaction and testing of *QYr*.*usw-5B* and *QYr*.*usw-7B* in the breeding panel

Phenotypic variation was observed in the infection assays for the breeding panel. For the seedling assays, the IT ranged from highly resistant (IT < 2) to highly susceptible (IT > 7) (S6 Table). The IT LSmeans for the disease assessment across the entire breeding panel ranged from 1.4 to 9.1 (S6 Table). The correlation among the infection assays using FC, W009 and W015 was high, with Pearson’s correlation coefficients larger than 0.77 (S7 Table). Under field conditions, DS ranged from 0 to 80% in 2013 and from 0 to 90% in 2014 in Mexico (S8 Table). DS and AUDPC were highly correlated between the two years (*r*^*2*^ ≥ 0.90). The adult plant resistance observed in 2013 and 2014 was moderately correlated with seedling resistance, with correlation coefficients ranging from 0.58 to 0.71.

**Fig 4:**
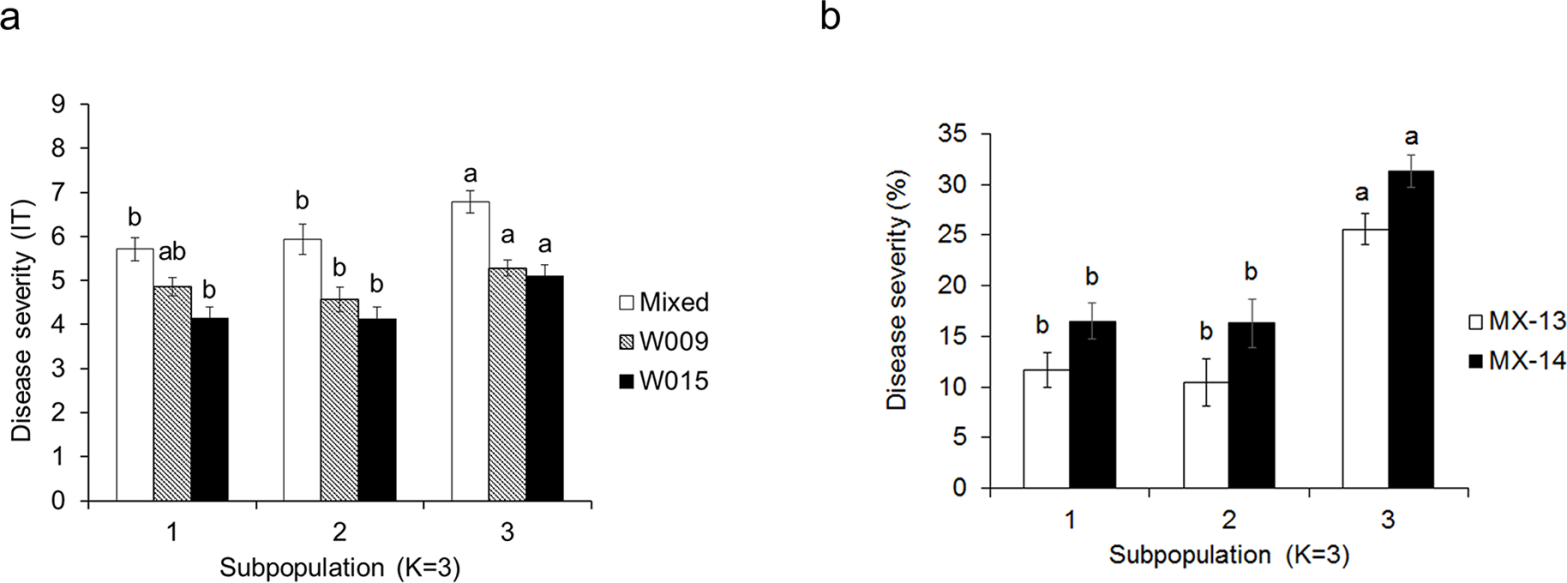
Mean strip rust infection in the breeding panel. (a) LSMeans for seedling disease severity of lines from three subpopulations that were inoculated with FC, W009, and W015. (b) LSMeans for adult plant disease severity of lines from three subpopulations, based on final ratings in 2013 (MX-13) and 2014 (MX-14). Bars that do not share a letter differ significantly (Fisher’s LSD, *P <* 0.05). Data are presented as mean ± standard error.

The 92 accessions were clustered into three subpopulations (SP1, SP2 and SP3) based on geographic distribution, phylogenetic relationship, and Bayesian clustering (S1 Fig) [54]. The first subpopulation, SP1, was comprised of 33 varieties from diverse origins, SP2 of 18 Italian accessions, and SP3 of 41 varieties from North America (Fig 4A). The LSmeans (from seedling and field experiments) across all cultivars within a subpopulation then were compared. The LSmeans for stripe rust resistance of SP3 (North American accessions) were significantly (*P <* 0.05) more susceptible compared to SP1 and SP2 (Fig 4A and 4B). The accessions in SP1 and SP2 had a more balanced distribution for resistance and susceptibility against stripe rust, and the average DS was not significantly different between SP1 and SP2. Similar trends were observed in the seedling and adult plant resistance.

To facilitate QTL testing, the breeding population was genotyped with the 90K iSelect SNP array (S9 Table; S2 Fig; S1 Appendix). The major locus *QYr*.*usw-7B* from the DH population was identified within a 3.0 cM region near the telomere of chromosome 7B in the breeding panel. Fifteen SNPs and one SSR marker were associated with stripe rust in some or all of the datasets (S10 Table). The proportion of phenotypic variance explained by the QTL when inoculated with W009, W015, and the FC ranged from 17.1% to 32.3% for seedling resistance, while for field resistance, it ranged from 11.6% to 22.6%. The *QYr*.*usw-5B* QTL identified in the DH population was not found in the breeding panel. The alleles at the marker *wsnp_Ku_c4427_8029592* were nearly fixed in most lines in this panel. Ninety-five percent of the lines studied carried the same allele as W9262-260D3 (resistant genotype from the DH population) at marker *wsnp_Ku_c4427_8029592* (peak marker of *QYr*.*usw-5B*) and five carried the same genotype as Kofa (susceptible genotype from the DH population). Lines carrying the susceptible genotype were all highly susceptible to stripe rust at all stages. Average disease scores placed these five individuals within the top 25% of the most susceptible lines; Mexa (IT = 6.7), Kofa (IT = 6.9), Westbred (IT = 7.2), Ocotillo (IT = 7.3) and Pathfinder (IT = 7.6). Mexa, Kofa, Westbred and Ocotillo also carried the susceptible genotype (same as Kofa) at the *QYr*.*usw-7B* locus. The only exception was Pathfinder, which had the resistant genotype (same as W9262-260D3) at *QYr*.*usw-7B*, but was still among the most severely affected by stripe rust among these five genotypes.

### Identification of gene candidates

Gene candidates for *QYr*.*usw-5B* and *QYr*.*usw-7B* were identified by mapping the markers for the QTL to the available genome sequence of WEW. *QYr*.*usw-5B* mapped to 3.63 Mbp interval ranging from 681.19 Mbp (*RAC875_c38873_1118*) to 684.82 Mbp (*wsnp_Ku_c4427_8029592*) on chromosome 5B. This interval contains 67 genes, of which 36 have high confidence annotations (Table 2). Two of these genes, *TRIDC5BG077260* and *TRIDC5BG077630*, are annotated as leucine-rich repeat receptor-like protein kinases, which have a role in pathogen recognition and disease resistance. Similarly, *QYr*.*usw-7B* mapped to a very small 267 kbp interval ranging from 719.49 Mbp (*BS00075300_51*) to 719.75 Mbp (*BS00003929_51*) on chromosome 7B. This interval contains seven genes, of which only four have high confidence annotations (Table 2). Two of the genes within *QYr*.*usw-7B*, *TRIDC7BG070820* and *TRIDC7BG070830*, are annotated as disease resistance proteins with nucleotide-binding site leucine-rich repeat domains.

**Table 2:** Gene candidates for *QYr*.*usw-5B* and *QYr*.*usw-7B* from the WEW genome.

Chr.	Position (Mbp)	Gene accession	Gene descriptor
5B	681.19	*TRIDC5BG077010*	actin 7
5B	681.19	*TRIDC5BG077030*	Myb/SANT-like DNA-binding domain protein
5B	681.42	*TRIDC5BG077040*	TCP family transcription factor
5B	681.43	*TRIDC5BG077050*	Cytochrome P450 superfamily protein
5B	681.43	*TRIDC5BG077060*	RNA-binding protein 1
5B	681.5	*TRIDC5BG077070*	unknown function
5B	681.5	*TRIDC5BG077080*	photosystem II reaction center PSB28 protein
5B	681.53	*TRIDC5BG077100*	Harpin-induced protein 1 containing protein, expressed
5B	681.54	*TRIDC5BG077120*	Harpin-induced protein 1 containing protein, expressed
5B	681.6	*TRIDC5BG077130*	Dehydrogenase/reductase SDR family member 4
5B	681.64	*TRIDC5BG077140*	IAA-amino acid hydrolase ILR1-like 3
5B	681.65	*TRIDC5BG077150*	IAA-amino acid hydrolase ILR1-like 4
5B	681.66	*TRIDC5BG077160*	IAA-amino acid hydrolase ILR1-like 4
5B	681.75	*TRIDC5BG077210*	mRNA-decapping enzyme-like protein
5B	681.8	*TRIDC5BG077230*	cellulose synthase 6
5B	681.89	*TRIDC5BG077260*	Leucine-rich repeat receptor-like protein kinase
5B	681.95	*TRIDC5BG077270*	ABC transporter G family member 45
5B	682.01	*TRIDC5BG077280*	nicotinate phosphoribosyltransferase 1
5B	682.1	*TRIDC5BG077290*	Pentatricopeptide repeat-containing protein
5B	682.1	*TRIDC5BG077300*	Eukaryotic translation initiation factor 4E-1
5B	682.11	*TRIDC5BG077310*	Eukaryotic translation initiation factor 4E-1
5B	682.11	*TRIDC5BG077320*	U3 small nucleolar RNA-associated protein 25
5B	682.21	*TRIDC5BG077330*	basic helix-loop-helix (bHLH) DNA-binding
5B	682.97	*TRIDC5BG077350*	unknown function
5B	683.63	*TRIDC5BG077430*	Bax inhibitor-1 family protein
5B	683.73	*TRIDC5BG077450*	Ubiquinone biosynthesis monooxygenase COQ6
5B	683.74	*TRIDC5BG077470*	E3 ubiquitin-protein ligase RNF170
5B	683.75	*TRIDC5BG077490*	Mitochondrial transcription termination factor
5B	684.23	*TRIDC5BG077530*	HXXXD-type acyl-transferase family protein
5B	684.25	*TRIDC5BG077540*	Subtilase family protein
5B	684.31	*TRIDC5BG077550*	ammonium transporter 2
5B	684.33	*TRIDC5BG077560*	Phosphoglycerate mutase family protein
5B	684.45	*TRIDC5BG077570*	Post-GPI attachment to proteins factor 3
5B	684.47	*TRIDC5BG077590*	Protein kinase superfamily protein
5B	684.49	*TRIDC5BG077600*	unknown function
5B	684.49	*TRIDC5BG077610*	OJ991214_12.8 protein
5B	684.63	*TRIDC5BG077630*	LRR receptor-like serine/threonine-protein kinase GSO1
5B	684.81	*TRIDC5BG077670*	Calmodulin-binding transcription activator 2
7B	719.48	*TRIDC7BG070820*	Disease resistance protein
7B	719.56	*TRIDC7BG070830*	Disease resistance protein (CC-NBS-LRR class) family
7B	719.67	*TRIDC7BG070850*	undescribed protein
7B	719.75	*TRIDC7BG070880*	RING/U-box superfamily protein

Exome sequencing identified 642 sequence variants, SNPs and small insertions or deletions (InDels), between Kofa and W9262-260D3 in *QYr*.*usw-5B* and *QYr*.*usw-7B* (S2 Appendix). While most of the variants identified were in intergenic regions and introns, which may have a role in regulating gene expression, 78 were within the coding sequences. Fifteen genes within *QYr*.*usw-5B* had sequence variation in their coding sequence, including InDels in the genes *TRIDC5BG077150* (IAA-amino acid hydrolase ILR1-like 4) and *TRIDC5BG077600* (unknown function) that are predicted to cause a shift in the reading frame and have a major effect on gene function. Variation was also detected within three genes from *QYr*.*usw-7B*, including predicted disease resistance genes *TRIDC7BG070820* and *TRIDC7BG070830*, which had four missense mutations each (S2 Appendix). All four missense variants for *TRIDC7BG070820* occurred within InterPro predicted leucine-rich repeat or nucleotide binding domains. Two out of four missense variants for *TRIDC7BG070830* occurred in InterPro predicted leucine-rich repeat or nucleotide binding domains, while the remaining two were part of the leucine-rich repeat unintegrated signature (S2 Appendix).

## Discussion

Stripe rust, a destructive disease of wheat occurring worldwide, can cause complete yield loss in extreme cases. In recent years, regional epidemics have occurred in North America (particularly the Pacific North-West), East Asia, South Asia, Australia, and East Africa [3]. In Canada, stripe rust has appeared more frequently in regions east of the Rocky Mountains, with epidemics occurring in southern Alberta and Saskatchewan in 2006, 2010 and 2011 [6]. Given the increased incidence of stripe rust and the limited number of effective disease resistance genes characterized in durum wheat, additional effort is required to identify new resistance genes that can be combined to mitigate the breakdown of currently available resistance [3, 19, 20]. In this study, we identified QTL on chromosomes 5B and 7B that confer resistance to stripe rust in durum wheat. The QTL on chromosome 5B was detected only in the DH population at the seedling stage. A second QTL, on chromosome 7B, was identified through QTL mapping of both seedling evaluations under controlled conditions and adult plant field trials in the DH population, and was also identified in global breeding lines.

### Genomic regions associated with stripe rust resistance on chromosome 7B

The effect of *QYr*.*usw-7B* on chromosome 7B in the DH population was confirmed through the discovery of a QTL at the same position in our breeding panel (S10 Table). The experimental evidence presented here suggests that this QTL contains a broad-spectrum resistance gene that is effective against multiple races of stripe rust, and is effective at all stages of plant development. *QYr*.*usw-7B* maps to the region on chromosome 7B known to harbor *Yr67* [50, 55] and *YrZH84* [56]. According to previous studies, 78 officially named resistance genes (*Yr1* to *Yr78*) and many temporarily designated genes (www.ars.usda.gov and www.shigen.nig.ac.jp) have been identified. Of the resistance genes currently known, *Yr2*, *Yr6*, *Yr39*, *Yr52*, *Yr59*, *YrZH84*, *Yr67* and *YrMY37* are located on chromosome 7B. The genes *Yr39*, *Yr52* and *Yr59* are adult plant resistance genes, which are not effective at the seedling stage and are therefore unlikely candidates for *QYr*.*usw-7B* [57]. Although *Yr2*, *Yr6*, *Yr67*, *YrZH84* and *YrMY37* could qualify as candidates because of their effectiveness at all growth stages, the differential lines carrying *Yr2* and *Yr6* were not effective against W009 and W015 [13]; therefore, *QYr*.*usw-7B* is functionally distinct from *Yr2* and *Yr6*. *YrMY37* was also ruled out as a candidate corresponding to the QTL we investigated because it is located on chromosome 7B close to the centromere [58], while *QYr*.*usw-7B* is near the distal end of chromosome 7B. *Yr67* (previously named *YrC591*) [50, 55] and *YrZH84* [56] are also all-stage dominant resistance genes mapped to the telomeric region of chromosome 7B. Lines carrying *Yr67* and *YrZH84* had different reactions to a panel of *Pst* isolates, indicating they are in fact distinct resistance genes [59]. Both genes share one significant flanking SSR marker with *QYr*.*usw-7B*, namely, *Xcfa2040-7B*. The relationship among *QYr*.*usw-7B*, *Yr67* and *YrZH84* needs further study, with allelism testing to estimate the genetic distance among them. However, both *Yr67* and *YrZH84* are outside of the physical interval for *QYr*.*usw-7B*, indicating that *QYr*.*usw-7B* may be a different gene (S3 Fig). The physical interval for *QYr*.*usw-7B* is very narrow and contains two genes annotated as pathogen receptors, each of which contained four missense mutations between Kofa and W9262-260D3 (Table 2, S2 Appendix). Additional research is required to validate the role of these gene candidates and the SNPs we identified, including their potential involvement in stripe rust resistance. Furthermore, linkage between *QYr*.*usw-7B* and other genes on chromosome 7B, including genes for yellow pigment and resistance to leaf rust, should also be investigated for breeding purposes [60].

### Genomic regions associated with stripe rust resistance on chromosome 5B

The QTL on chromosome 5B, *QYr*.*usw-5B*, was found to confer resistance to *Pst* isolates W009 and W015 in the Kofa/W9262 DH population. The QTL was flanked by *RAC875_c38873_1118* and *wsnp_Ku_c4427_8029592*, with the peak situated nearest to the latter marker. The QTL explained 25.1% and 11.0% of phenotypic variance in seedling resistance to W009 and W015, respectively. *QYr*.*usw-5B* showed strong epistatic interaction with *QYr*.*usw-7B* in the DH mapping population but was not identified in the breeding panel. Upon closer inspection, it was found that the susceptible allele at marker *wsnp_Ku_c4427_8029592*, the peak marker for *QYr*.*usw-5B*, was nearly absent in the breeding panel, which may have limited our ability to detect the QTL. The five lines that carried the susceptible allele all had severe disease ratings in all trials. The reduced representation of this allele in such a diverse set of lines suggests that breeding programs have selected for the resistant allele at this locus. In previous studies, seedling resistance genes *Yr19*, *Yr47*, and *Yr74* were also found to be located on chromosome 5B. *Yr47* was identified as a seedling resistance gene located on chromosome 5BS [61], whereas *QYr*.*usw-5B* is located on 5BL. Recently, *Yr74* was identified as an all stage resistance gene in the Australian hexaploid wheat Avocet R (AvR)-AUS 90660; however, there is no information regarding this gene in durum wheat and the assocciated DArT-Seq markers are unavailable for comparative analysis to *QYr*.*usw-5B* [62]. *Yr19* is a dominant resistance gene located on chromosome 5B discovered in an F_2_ population from crosses of disomic aneuploidy lines of Chinese Spring [63], but no marker information is available for *Yr19*. The relationship between *QYr*.*usw-5B*, *Yr74*, and *Yr19* is unknown at this time and warrants further investigation. Comparison of QTL across populations can be facilitated by using a common set of markers or a common reference genome sequence. Our study provides a physical location for *QYr*.*usw-5B* in an available tetraploid reference genome and lists gene candidates from the interval as well as sequence variation within genes and intergenic regions that may be involved in stripe rust resistance. However, the sequence variation identified in this study using exome capture sequencing does not fully capture all genes and their variants; therefore, additional variants within genes and intergenic regions likely exist between Kofa and W9262-260D3 for both *QYr*.*usw-5B* and *QYr*.*usw-7B* that could be identified by a more comprehensive sequence strategy. Additional research is also required to determine if the variation within these gene candidates are involved in stripe rust resistance.

### Interaction between rust resistance QTL on chromosomes 5B and 7B

The full expression of resistance in the DH population requires both *QYr*.*usw-5B* and *QYr*.*usw-7B* (Table 1). The statistical analysis indicated that the epistatic interaction between *QYr*.*usw-5B* and *QYr*.*usw-7B* was significant (*P <* 0.01), and genes are most effective when stacked. Epistatic interactions that involve rust resistance genes have been documented in other studies. For example, an epistatic interaction was identified between a QTL for stripe rust resistance on chromosome 2AS and QTL on chromosome 6AL, which resulted in some inbred lines that had resistance that was equal to or greater than the resistant parent [64]. Similarly, an epistatic interaction was identified between QTL that conferred resistance to stem rust resistance in a DH population derived from the Canadian wheat cultivars AC Cadillac and Carberry [65]. Epistatic interactions were also observed between multiple pairs of QTL in other studies, including QTL on chromosomes 4B and 5B, 4B and 7B, 5B and 6D, 6D and 3B, and 6D and 7B. In research conducted by Yu et al. [66] on stem rust resistance of CIMMYT spring wheat, multiple significant pairwise QTL interactions were detected. The *Sr2* locus on chromosome 3BS and the *wPt1859* locus on 7DL interacted with other loci on the same chromosome and with markers on chromosome 6B. Interactions also involved loci on chromosomes 1B, 4A and 2B. Yu et al. [67] also conducted research on stem rust resistance of winter wheat to Ug99, and showed that multiple loci were involved in the QTL interaction, including loci on chromosomes 3BS, 6BS, 2BS and 7DS. This suggests that complex genetic control for adult plant resistance to stem rust isolate Ug99 exists in winter wheat. Kumar et al. [68] studied leaf rust and stripe rust resistance in the International Triticeae Mapping Initiative (ITMI) population and identified eight QTL interactions for each trait, as well as and epistatic-QTL interaction between loci on chromosomes 1D and 3B. Together, epistatic interactions between QTL relating to resistance to rust pathogens is common, and the interaction we identified between QTL on chromosomes 7B and 5B will be actively incorporated into breeding programs to enhance resistance to stripe rust in Canadian germplasm.

### Germplasm from the breeding panel can be used in future crosses to enhance resistance to stripe rust

Genetic exchange among breeding programs is critical to widen the genetic base of any program in general, and to enhance stripe rust resistance in particular. The majority of Canadian durum germplasm were susceptible to stripe rust at the seedling stage; the percentage of susceptible lines was 100% for FC, 75% for W009, and 83% for W015. In the Mexican field trials, percentage of the Canadian lines that displayed adult plant DS greater than 20% was 46% in 2013 and 57% in 2014. Some Canadian lines that were susceptible at the seedling stage were resistant at the adult plant stage, such as Strongfield, 9661-AF1D, 9661-CA5E, D24-1773, DT513, DT710, and DT711. Other resistant or moderately susceptible lines at the seedling stage were also highly resistant at the adult plant stage, including AC Avonlea, Napoleon, CDC Verona, DT696, DT705, DT707, and Kyle. Although stripe rust is not currently a common disease of durum wheat in Canada, the recent epidemics in hexaploid wheat in AB and SK may indicate a trend towards increased disease pressure and a need to develop Canadian durum cultivars with increased resistance. In the breeding panel, some lines from other breeding programs were highly resistant at both seedling and adult plant stages, such as Buck Ambar (Argentina), Carioca (France), Durabon (Germany), D-73-15 (Iran), Arcobaleno (Italy), Ciccio (Italy), Iride (Italy), Parsifal (Italy), Tresor (Italy), DHTON 1 (Morocco), Arrivato (New Zealand), CFR5001 (New Zealand), CRDW17 (New Zealand), Altar-Aos (Spain), and Gallareta (Spain). Together, the identification of *Pst* resistance genes in both domestic and foreign cultivars, and the development of usable molecular markers linked to their resistance, will be invaluable to aid breeding efforts aimed at preserving durum wheat as a global crop that is both viable and competitive.

## Conclusions

We used genetic mapping to detect two QTL (*QYr*.*usw-5B* and *QYr*.*usw-7B*) that conferred stripe rust resistance in a DH population, one of which (*QYr*.*usw-7B*) was also identified in a diverse breeding panel. The physical locations of the QTL were determined in the WEW genome and candidate resistance genes and genetic variations within the interval were identified. Within the DH population, a strong epistatic interaction was observed between *QYr*.*usw-5B* and *QYr*.*usw-7B*. Combining the major QTL from this study with other effective resistance genes via marker assisted selection could be applied in future breeding programs to develop durable resistance to stripe rust. The 90K wheat SNP assay greatly facilitated the identification of the QTL in the DH population and provided a standard set of SNP markers that could be used to pyramid the QTL we investigated with those identified from other studies.

## Supporting information

S1 Fig Population structure for the breeding panel. (a) Consensus phylogenetic tree constructed using Rogers’ Euclidean distance for the 92 durum wheat cultivars; the color strip represents the composition of three sub-populations. (b) Principal component analysis where each dot represents one of the 92 lines of the breeding population in a space formed by Prin1, Prin2 and Prin3; the dots were colored according to model-based Bayesian clustering analysis using STRUCTURE V2.3.4.
(DOCX)

S2 Fig Genotyping and association analysis of the breeding panel. (a) Linkage disequilibrium (*r*^*2*^) decay plot of pair-wise markers as a function of genetic distance (cM) for the breeding panel. The fitted curve (red) shows the expected LD decay between adjacent 90K iSelect SNP array markers based on a nonlinear regression model. The critical *r*^*2*^ value (dashed line) is the 95% quantile of *r*^*2*^ value of unlinked SNP markers. (b) Quantile-Quantile (Q-Q) plot of three different models for population structure and kinship. The expected *P*-values were plotted against observed *P*-values for each SNP, based on three different models: the naïve model (blue), GLM with three sub-populations (red), and MLM with three sub-populations and kinship (green). The diagonal reference line (dashed line) represented the null hypothesis of no association. The Q-Q plot was based on the seedling test of isolate W009, which is the most representative Q-Q plot among all phenotypic data.
(DOCX)

S3 Fig Physical map of *QYr*.*usw-7B* in the wild emmer wheat genome. Markers from *QYr*.*usw-7B*, *YrZH84*, and *YrC591*/*Yr67* were mapped to the wild emmer wheat genome using GMAP. *QYr*.*usw-7B* (left) is positioned proximal to *YrZH84* (middle) and *YrC591* or *Yr67* (right). QTL regions are highlighted by black shading. Positions of flanking markers, in Mbp, are indicated on the left side of each map.
(DOCX)

S1 Table Origin and pedigree information for the breeding population. (DOCX)

S2 Table Experimental design of seedling disease resistance assays. (DOCX)

S3 Table Experimental design of adult disease resistance assays. (DOCX)

S4 Table The sequence of filters to remove false-positive 90k iSelect SNP calls in GenomeStudio. (DOCX)

S5 Table Mapping statistics for the DH population genetic map. (DOCX)

S6 Table LSMeans of seedling stripe rust reaction to FC, W009, and W015 (IT: 0–9) within the breeding panel. (DOCX)

S7 Table Pearson correlations between seedling infection type for the breeding population inoculated with FC, W009 and W015. (DOCX)

S8 Table LSMeans for the final recording and area under the disease progress curve (AUDPC) for all recordings of adult plant resistance for the breeding panel evaluated in Mexico 2013 and 2014. (DOCX)

S9 Table Polymorphic SNP markers in the breeding population. (DOCX)

S10 Table Summary of significant markers located on chromosome 7B associated with stripe rust resistance in the breeding panel, including seedling reaction to FC, single isolates W009 and W015 and adult plant resistance reaction in Mexico in 2013 and 2014. (DOCX)

S1 Appendix 90K wheat SNP assay marker calls. (XLSX)

S2 Appendix Exome sequence variants and their predicted effects on gene function. (XLSX)
