# Supplementary material for: Genetic analysis of resistance to stripe rust in durum wheat (Triticum turgidum L. var. durum)
Source: PLoS One. 2018 Sep 19;13(9):e0203283. doi: 10.1371/journal.pone.0203283 (PMC6145575; doi:10.1371/journal.pone.0203283)
Supplement: S1 Fig — (a) Consensus phylogenetic tree constructed using Rogers’ Euclidean distance for the 92 durum wheat cultivars; the color strip represents the composition of three sub-populations. (b) Principal component analysis where each dot represents one of the 92 lines of the breeding population in a space formed by Prin1, Prin2 and Prin3; the dots were colored according to model-based Bayesian clustering analysis using STRUCTURE V2.3.4. (DOCX) [file pone.0203283.s001.docx]

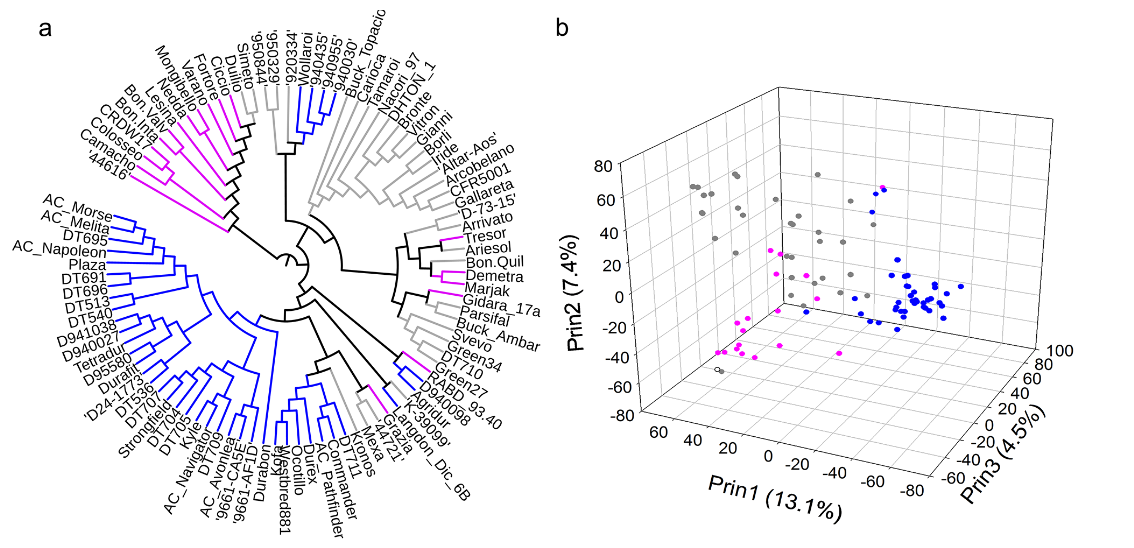


# S1 Fig. Population structure for the breeding panel. (a) Consensus phylogenetic tree constructed using Rogers’ Euclidean distance for the 92 durum wheat cultivars; the color strip represents the composition of three sub-populations. (b) Principal component analysis where each dot represents one of the 92 lines of the breeding population in a space formed by Prin1, Prin2 and Prin3; the dots were colored according to model-based Bayesian clustering analysis using STRUCTURE V2.3.4.
