# Supplementary material for: Genetic analysis of resistance to stripe rust in durum wheat (Triticum turgidum L. var. durum)
Source: PLoS One. 2018 Sep 19;13(9):e0203283. doi: 10.1371/journal.pone.0203283 (PMC6145575; doi:10.1371/journal.pone.0203283)
Supplement: S2 Fig — (a) Linkage disequilibrium (r2) decay plot of pair-wise markers as a function of genetic distance (cM) for the breeding panel. The fitted curve (red) shows the expected LD decay between adjacent 90K iSelect SNP array markers based on a nonlinear regression model. The critical r2 value (dashed line) is the 95% quantile of r2 value of unlinked SNP markers. (b) Quantile-Quantile (Q-Q) plot of three different models for population structure and kinship. The expected P-values were plotted against observed P-values for each SNP, based on three different models: the naïve model (blue), GLM with three sub-populations (red), and MLM with three sub-populations and kinship (green). The diagonal reference line (dashed line) represented the null hypothesis of no association. The Q-Q plot was based on the seedling test of isolate W009, which is the most representative Q-Q plot among all phenotypic data. (DOCX) [file pone.0203283.s002.docx]

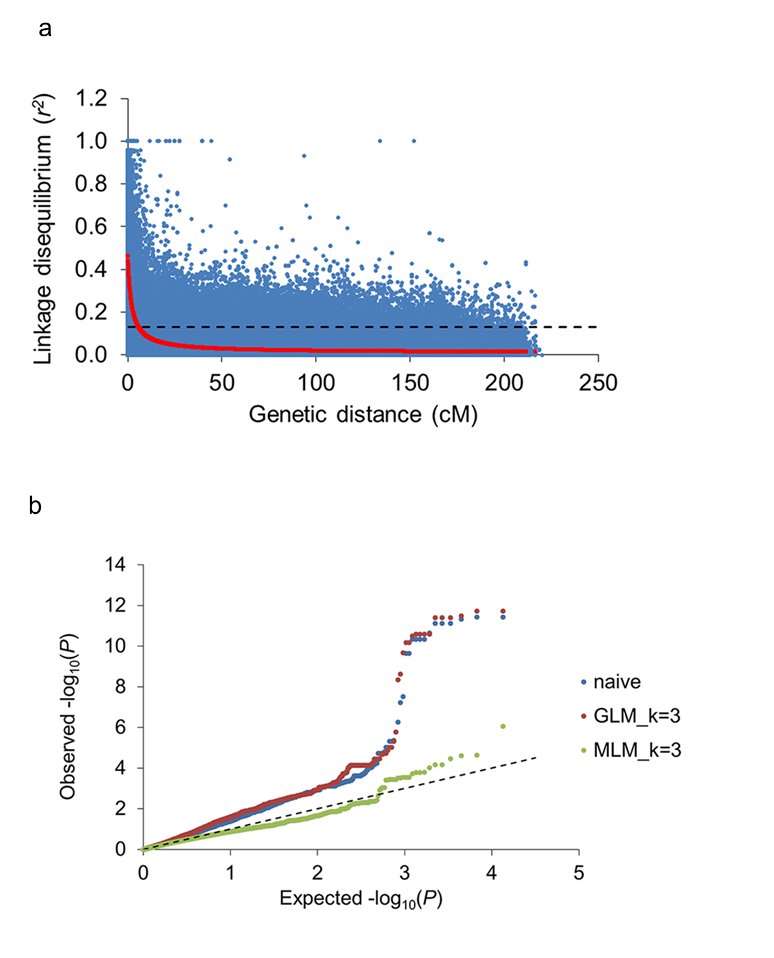


# S2 Fig. Genotyping and association analysis of the breeding panel. (a) Linkage disequilibrium (*r^2^*) decay plot of pair-wise markers as a function of genetic distance (cM) for the breeding panel. The fitted curve (red) shows the expected LD decay between adjacent 90K iSelect SNP array markers based on a nonlinear regression model. The critical *r^2^* value (dashed line) is the 95% quantile of *r^2^* value of unlinked SNP markers. (b) Quantile-Quantile (Q-Q) plot of three different models for population structure and kinship. The expected *P*-values were plotted against observed *P*-values for each SNP, based on three different models: the naïve model (blue), GLM with three sub-populations (red), and MLM with three sub-populations and kinship (green). The diagonal reference line (dashed line) represented the null hypothesis of no association. The Q-Q plot was based on the seedling test of isolate W009, which is the most representative Q-Q plot among all phenotypic data.
