# Supplementary material for: Genetic analysis of resistance to stripe rust in durum wheat (Triticum turgidum L. var. durum)
Source: PLoS One. 2018 Sep 19;13(9):e0203283. doi: 10.1371/journal.pone.0203283 (PMC6145575; doi:10.1371/journal.pone.0203283)
Supplement: S3 Fig — Markers from QYr.usw-7B, YrZH84, and YrC591/Yr67 were mapped to the wild emmer wheat genome using GMAP. QYr.usw-7B (left) is positioned proximal to YrZH84 (middle) and YrC591 or Yr67 (right). QTL regions are highlighted by black shading. Positions of flanking markers, in Mbp, are indicated on the left side of each map. (DOCX) [file pone.0203283.s003.docx]

#
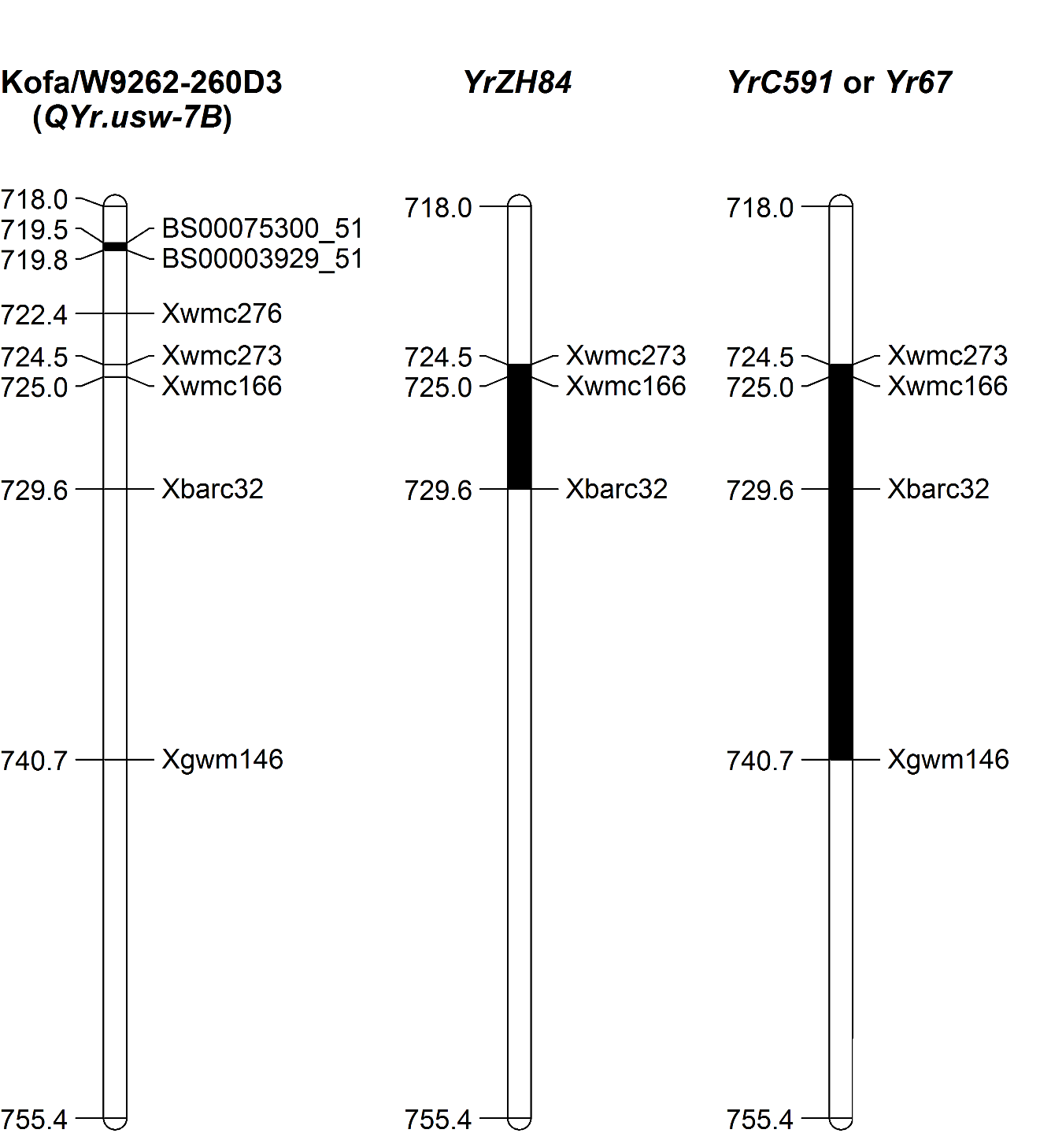


# S3 Fig. Physical map of *QYr.usw-7B* in the wild emmer wheat genome. Markers from *QYr.usw-7B*, *YrZH84*, and *YrC591*/*Yr67* were mapped to the wild emmer wheat genome using GMAP. *QYr.usw-7B* (left) is positioned proximal to *YrZH84* (middle) and *YrC591* or *Yr67* (right). QTL regions are highlighted by black shading. Positions of flanking markers, in Mbp, are indicated on the left side of each map.
