# Supplementary material for: Genetic analysis of resistance to stripe rust in durum wheat (Triticum turgidum L. var. durum)
Source: PLoS One. 2018 Sep 19;13(9):e0203283. doi: 10.1371/journal.pone.0203283 (PMC6145575; doi:10.1371/journal.pone.0203283)
Supplement: S1 Table — (DOCX) [file pone.0203283.s004.docx]

# S1 Table Origin and pedigree information for the breeding population.

| Accession | Origin | Pedigree |
| --- | --- | --- |
| Bonaerance Inta Cumenay | Argentina | Unknown |
| Bonaerance Quilaco | Argentina | MAGH72//GS/AA///RABI//D21563/AA |
| Bonaerance Valverde | Argentina | GIORGIO//CAPELLI/YUMA |
| Buck Ambar | Argentina | Unknown |
| Buck Topacio | Argentina | Unknown |
| 920334 | Australia | 69850/ 86014 |
| 940030 | Australia | Unknown |
| 940435 | Australia | Unknown |
| 940955 | Australia | Unknown |
| 950329 | Australia | Unknown |
| 950844 | Australia | Unknown |
| Tamaroi | Australia | RUFF/FLAMINGO‐DW//MEXICALI‐75///SHEARWATER/56113/TAM‐1‐B‐17/KAMILAROI/56112/WELLS/56111//GUILLEMOT |
| Wollaroi | Australia | TAM‐1‐B‐17/(SIB)KAMILAROI//ROKEL(S)/(SIB)KAMILAROI |
| 9661-AF1D | Canada | W9262‐260D3/ARUBA//DT 662 |
| 9661-CA5E | Canada | W9262‐260D3/ARUBA//DT 662 |
| AC Avonlea | Canada | 8267‐AD2A/DT 61 |
| AC Melita | Canada | MEDORA/LLOYD |
| AC Morse | Canada | RL 7196/DT 610 |
| Napoleon | Canada | VIC/DT384//DT 471 |
| AC Navigator | Canada | KYLE/WESTBRED881 |
| AC Pathfinder | Canada | WESTBRED 881/DT 367 |
| Commander | Canada | W9260‐BK03/AC NAVIGATOR//AC PATHFINDER |
| D24-1773 | Canada | DT 520/D94078 |
| DT513 | Canada | DT 625/DT 612 |
| DT536 | Canada | D94350/D93108 |
| CDC Verona | Canada | D95253/D95116 |
| DT691 | Canada | DT618/ 8667‐D216C//DT 637 |
| DT695 | Canada | DT 471/2*KYLE |
| DT696 | Canada | DT618/DT 637//KYLE |
| DT704 | Canada | AC AVONLEA/DT 665 |
| DT705 | Canada | AC AVONLEA/DT 665 |
| DT707 | Canada | AC AVONLEA/DT 665 |
| DT709 | Canada | DT 674/DT 665 |
| DT710 | Canada | DT618/Green 27 |
| DT711 | Canada | Westbred 881/W9260‐BK03 |
| Kyle | Canada | 6962‐92‐8‐5/ 6965‐494‐ |
| Strongfield | Canada | AC Avonlea/DT 665 |
| Agridur | France | EDMORE//CIMMYT 303/CHANDUR |
| Ariesol | France | Unknown |
| Carioca | France | CID 479402 |
| RABD 93.40 | France | Unknown |
| Tetradur | France | EDMORE//CAPDUR/REGAL |
| Durabon | Germany | SIGNADUR/EDM//P 4312.86 |
| Durafit | Germany | Unknown |
| 44616 | Iran | Unknown |
| 44721 | Iran | Unknown |
| D-73-15 | Iran | Unknown |
| Arcobaleno | Italy | CHEN/ALTAR 84 |
| Bronte | Italy | BERILLO/LATINO |
| Ciccio | Italy | APPULO/VALNOVA//VALFORTE/PATRIZIO |
| Colosseo | Italy | CRESO/MEXA |
| Demetra | Italy | MESSAPIA/GIOIA |
| Duilio | Italy | CAPPELLI//ANHINGA/FLAMINGO |
| Fortore | Italy | CAPEITI 8/VALFORTE |
| Gianni | Italy | Unknown |
| Grazia | Italy | ISWRN‐21/VALSELVA |
| Iride | Italy | ALTAR 84/ARES‐SIB |
| Lesina | Italy | Unknown |
| Mongibello | Italy | TRINAKRIA/VALFORTE |
| Nedda | Italy | TRINAKRIA/VALFORTE |
| Parsifal | Italy | INRA92‐1/D81028 |
| Simeto | Italy | CAPEITI/VALNOVA |
| Svevo | Italy | SELEZIONE CIMMYT/ZENIT‐SIB |
| Tresor | Italy | AMBER‐DURUM/S‐22‐80 |
| Varano | Italy | CAPEITI 8/CRESO//CRESO///VALFORTE/TRINAKRIA |
| Green 27 | Mexico | STERNA‐DW 2/GRAVELOTE |
| Green 34 | Mexico | STERNA‐DW 2/GRAVELOTE |
| Nacori 97 | Mexico | ALTAR 84/CMH82A.1062//CD58230‐? |
| Vitron | Mexico | TURCHIA‐77///JORI‐SIB/ANHINGA‐SIB//FLAMINGO‐SIB |
| DHTON 1 | Morocco | Unknown |
| Gidara 17a | Morocco | Unknown |
| Marjak | Morocco | Unknown |
| Arrivato | New Zealand | Unknown |
| CFR5001 | New Zealand | Unknown |
| CRDW17 | New Zealand | Unknown |
| K-39099 | Russia | LV‐URAZOVSKII R‐N,VORONEZHSKAYA OBL |
| Altar-Aos | Spain | Unknown |
| Borli | Spain | Unknown |
| Camacho | Spain | Unknown |
| Gallareta | Spain | RUFF/FLAMINGO‐DW//MEXICALI‐75/3/SHEARWATER/4/? |
| Mexa | Spain | GDOVZ469///JO 1//61.130/LDS |
| D940027 | U.S. | D88104/D88207 |
| D940098 | U.S. | D88450/D87436 |
| D941038 | U.S. | D86117/D88289 |
| D95580 | U.S. | BELZER/D88058//D88276 |
| Durex | U.S. | AZ‐MFSRS‐86 |
| Kofa | U.S. | DICOCCUM ALPHA |
| Kronos | U.S. | APB MSFRS POP SEL (D03‐21) |
| Langdon | U.S. | LDN240/KHAPLI//LANGDON 308///MINDUM*3/VERNAL/4/VERNAL EMMER/3*MINDUM |
| Ocotillo | U.S. | Unknown |
| Plaza | U.S. | PLENTY/D8291 |
| Westbred881 | U.S. | WARD/WLS//CNDO/WCA///MEXI/WB1000 |
