# Supplementary material for: Genetic analysis of resistance to stripe rust in durum wheat (Triticum turgidum L. var. durum)
Source: PLoS One. 2018 Sep 19;13(9):e0203283. doi: 10.1371/journal.pone.0203283 (PMC6145575; doi:10.1371/journal.pone.0203283)
Supplement: S2 Table — (DOCX) [file pone.0203283.s005.docx]

# S2 Table Experimental design of seedling disease resistance assays.

| Population | Stripe rust isolate | Experimental design | No. of replications | Checks | |
| --- | --- | --- | --- | --- | --- |
|  |  |  |  | Susceptible | Resistant |
| DH | W009 | Alpha-lattice design | 3 | Kofa, Avocet | W9262-260D3 |
|  | W015 |  |  | Kofa, Avocet | W9262-260D3 |
| Validation | FC | Alpha-lattice design | 3 | Brigade, DT749 | Lillian, DT546 |
|  | W009 |  |  | Avocet, Brigade, DT749 | Lillian, DT546 |
|  | W015 |  |  | Avocet, Brigade, DT749 | Lillian, DT546 |
