# Supplementary material for: Genetic analysis of resistance to stripe rust in durum wheat (Triticum turgidum L. var. durum)
Source: PLoS One. 2018 Sep 19;13(9):e0203283. doi: 10.1371/journal.pone.0203283 (PMC6145575; doi:10.1371/journal.pone.0203283)
Supplement: S3 Table — (DOCX) [file pone.0203283.s006.docx]

# S3 Table Experimental design of adult disease resistance assays.

| Population | Year | Field Location | Experimental design | No. of replications | Checks | |
| --- | --- | --- | --- | --- | --- | --- |
|  |  |  |  |  | Susceptible | Resistant |
| DH | 2014 | Toluca, Mexico | RCBD | 3 | Kofa, Avocet, Westbred881 | W9262-260D3, Lillian |
| Validation | 2013 | Toluca, Mexico | RCBD | 3 | Avocet, Brigade | Lillian, DT546 |
|  | 2014 |  |  |  | Avocet, Brigade, DT532 | Lillian |
