# Supplementary material for: Genetic analysis of resistance to stripe rust in durum wheat (Triticum turgidum L. var. durum)
Source: PLoS One. 2018 Sep 19;13(9):e0203283. doi: 10.1371/journal.pone.0203283 (PMC6145575; doi:10.1371/journal.pone.0203283)
Supplement: S4 Table — (DOCX) [file pone.0203283.s007.docx]

# S4 Table The sequence of filters to remove false-positive 90k iSelect SNP calls in GenomeStudio.

| Steps | Filters | Description | Threshold to  keep markers | Num. of SNPs left |
| --- | --- | --- | --- | --- |
| 1 | Allele Freq. | Remove all monomorphic markers | Allele frequency < 1 | 48,248 |
| 2 | Cluster Sep. | Cluster separation – genotypic clusters separated into two discrete groups | Cluster Sep >= 0.2 | 47,830 |
| 3 | Call Freq. | Removal of SNP markers with > 5% missing data | Call Freq >= 0.95 | 36,813 |
| 4 | AB R Mean | Remove SNP markers with low signal intensity | AB R Mean >= 0.2 | 36,618 |
| 5 | AB T Mean | Remove SNP markers where AB clusters has shifted toward the AA or BB | 0.2 < AB T Mean < 0.8 | 35,175 |
| 6 | Minor Freq. | Removed markers with a minor allele frequency of less than 10% | Minor Freq >= 0.1 | 13,539 |
