# Supplementary material for: Genetic analysis of resistance to stripe rust in durum wheat (Triticum turgidum L. var. durum)
Source: PLoS One. 2018 Sep 19;13(9):e0203283. doi: 10.1371/journal.pone.0203283 (PMC6145575; doi:10.1371/journal.pone.0203283)
Supplement: S5 Table — (DOCX) [file pone.0203283.s008.docx]

# S5 Table Mapping statistics for the DH population genetic map.

| Chromosome | Length (cM) | Num. of markers unique mapped ^a^ | Density (Num. of markers/cM) | Max. gap (cM) | Num. of gaps > 10 cM |
| --- | --- | --- | --- | --- | --- |
| 1A | 126.6 | 41 | 0.32 | 22.87 | 4 |
| 1B | 214.6 | 168 | 0.78 | 14.06 | 1 |
| 2A | 151.4 | 108 | 0.71 | 8.85 | 0 |
| 2B | 199.7 | 179 | 0.90 | 9.57 | 0 |
| 3A | 143.0 | 61 | 0.43 | 24.96 | 4 |
| 3B | 220.0 | 126 | 0.57 | 20.64 | 1 |
| 4A | 164.1 | 91 | 0.55 | 13.48 | 1 |
| 4B | 141.9 | 87 | 0.61 | 6.90 | 0 |
| 5A | 195.5 | 94 | 0.48 | 14.75 | 1 |
| 5B | 246.3 | 181 | 0.73 | 7.08 | 0 |
| 6A | 156.2 | 100 | 0.64 | 10.88 | 1 |
| 6B | 201.4 | 145 | 0.72 | 8.33 | 0 |
| 7A | 207.7 | 126 | 0.61 | 7.99 | 0 |
| 7B | 271.3 | 181 | 0.67 | 7.58 | 0 |
| Whole genome | 2639.7 | 1688 | 0.64 | 24.96 | 13 |

^a^ 10,875 polymorphic markers were mapped and the number of unique map positions is presented.
