# Supplementary material for: Genetic analysis of resistance to stripe rust in durum wheat (Triticum turgidum L. var. durum)
Source: PLoS One. 2018 Sep 19;13(9):e0203283. doi: 10.1371/journal.pone.0203283 (PMC6145575; doi:10.1371/journal.pone.0203283)
Supplement: S6 Table — (DOCX) [file pone.0203283.s009.docx]

#

# S6 Table LSMeans of seedling stripe rust reaction to FC, W009, and W015 (IT: 0-9) within the breeding panel.

| Accession | FC | | W009 | | W015 | |
| --- | --- | --- | --- | --- | --- | --- |
|  | Scoring_1 | Scoring_2 | Scoring_1 | Scoring_2 | Scoring_1 | Scoring_2 |
| Bonaerance Inta Cumenay | 5.1 | 5.7 | 2.6 | 3.4 | 4.5 | 4.6 |
| Bonaerance Quilaco | 7.5 | 8.1 | 6.4 | 7.1 | 6.9 | 7.0 |
| Bonaerance Valverde | 6.0 | 7.3 | 5.6 | 7.2 | 3.9 | 5.3 |
| Buck Ambar | 1.6 | 1.7 | 1.8 | 1.8 | 2.1 | 2.1 |
| Buck Topacio | 3.5 | 5.7 | 3.4 | 4.2 | 4.9 | 5.4 |
| 920334 | 6.5 | 8.1 | 5.4 | 6.7 | 5.4 | 5.9 |
| 940030 | 8.4 | 8.3 | 6.3 | 7.1 | 6.6 | 6.7 |
| 940435 | 4.5 | 6.3 | 5.1 | 6.9 | 3.9 | 4.2 |
| 940955 | 6.3 | 7.9 | 5.2 | 6.7 | 4.0 | 4.4 |
| 950329 | 6.6 | 8.1 | 6.0 | 7.7 | 4.9 | 5.6 |
| 950844 | 7.4 | 8.4 | 6.9 | 7.0 | 5.1 | 5.7 |
| Tamaroi | 4.7 | 6.0 | 4.5 | 4.7 | 3.9 | 4.1 |
| Wollaroi | 4.4 | 6.9 | 3.7 | 5.0 | 3.2 | 3.7 |
| 9661-AF1D | 6.3 | 7.2 | 5.0 | 5.9 | 5.1 | 5.2 |
| 9661-CA5E | 5.1 | 6.3 | 4.2 | 4.3 | 3.4 | 3.6 |
| AC Avonlea | 4.7 | 5.0 | 4.0 | 4.5 | 3.7 | 3.9 |
| AC Melita | 6.4 | 7.9 | 5.4 | 6.1 | 4.0 | 4.5 |
| AC Morse | 7.4 | 8.3 | 6.2 | 7.1 | 6.6 | 6.8 |
| Napoleon | 4.4 | 4.3 | 2.2 | 2.2 | 4.2 | 4.7 |
| AC Navigator | 7.6 | 8.8 | 7.7 | 7.6 | 6.6 | 7.3 |
| AC Pathfinder | 7.8 | 8.9 | 6.5 | 7.6 | 5.8 | 6.1 |
| Commander | 5.4 | 7.5 | 6.1 | 6.6 | 6.4 | 6.8 |
| D24-1773 | 4.5 | 5.9 | 4.1 | 5.3 | 4.0 | 4.6 |
| DT513 | 5.5 | 6.9 | 3.4 | 4.6 | 3.6 | 4.2 |
| DT536 | 6.7 | 8.9 | 6.9 | 7.3 | 6.9 | 6.9 |
| CDC Verona | 3.6 | 5.2 | 3.1 | 3.6 | 4.7 | 5.7 |
| DT691 | 5.4 | 5.9 | 3.4 | 3.3 | 4.4 | 4.2 |
| DT695 | 5.9 | 5.8 | 2.6 | 2.5 | 3.9 | 3.9 |
| DT696 | 4.4 | 5.8 | 2.7 | 3.4 | 5.7 | 6.1 |
| DT704 | 7.2 | 8.2 | 6.2 | 7.0 | 6.3 | 6.5 |
| DT705 | 4.4 | 5.1 | 3.1 | 3.6 | 3.4 | 4.2 |
| DT707 | 4.2 | 4.3 | 4.3 | 4.2 | 3.8 | 4.3 |
| DT709 | 7.2 | 8.0 | 5.9 | 6.8 | 5.5 | 6.1 |
| DT710 | 5.6 | 6.5 | 3.8 | 4.3 | 3.6 | 3.9 |
| DT711 | 7.1 | 7.6 | 5.6 | 6.5 | 5.2 | 6.3 |
| Kyle | 4.4 | 5.4 | 3.7 | 4.7 | 3.7 | 4.8 |
| Strongfield | 4.6 | 6.4 | 4.0 | 4.5 | 4.5 | 5.2 |
| Agridur | 8.4 | 8.8 | 7.3 | 7.3 | 6.7 | 6.5 |
| Ariesol | 5.1 | 6.3 | 4.5 | 4.5 | 4.0 | 4.5 |
| Carioca | 2.0 | 1.8 | 1.8 | 1.6 | 1.8 | 2.2 |
| RABD 93.40 | 4.0 | 5.3 | 3.1 | 4.3 | 2.9 | 3.1 |
| Tetradur | 3.6 | 4.8 | 3.1 | 3.7 | 3.2 | 3.0 |
| Durabon | 3.8 | 3.5 | 2.5 | 2.4 | 3.0 | 3.2 |
| Durafit | 7.1 | 8.3 | 7.0 | 7.3 | 6.0 | 6.8 |
| 44616 | 4.9 | 6.4 | 4.0 | 4.1 | 3.3 | 3.5 |
| 44721 | 5.2 | 6.9 | 5.3 | 6.3 | 3.1 | 3.7 |
| D-73-15 | 2.7 | 2.4 | 2.4 | 2.6 | 2.7 | 2.9 |
| Arcobaleno | 2.1 | 2.3 | 2.1 | 2.2 | 2.0 | 2.1 |
| Bronte | 7.0 | 7.8 | 6.0 | 6.4 | 5.8 | 6.1 |
| Ciccio | 3.2 | 3.8 | 2.7 | 3.0 | 3.3 | 3.6 |
| Colosseo | 4.7 | 6.3 | 5.1 | 6.0 | 5.2 | 5.5 |
| Demetra | 3.6 | 5.8 | 6.0 | 6.8 | 5.0 | 5.1 |
| Duilio | 5.8 | 6.9 | 4.1 | 5.7 | 4.1 | 4.3 |
| Fortore | 3.6 | 6.7 | 3.4 | 3.7 | 2.8 | 3.3 |
| Gianni | 7.8 | 7.9 | 6.4 | 6.9 | 5.7 | 5.8 |
| Grazia | 2.4 | 2.7 | 2.6 | 2.7 | 3.7 | 4.0 |
| Iride | 2.5 | 2.7 | 2.3 | 2.1 | 2.0 | 2.1 |
| Lesina | 5.0 | 7.7 | 4.4 | 5.5 | 3.9 | 4.5 |
| Mongibello | 6.3 | 7.3 | 5.1 | 6.3 | 4.9 | 5.3 |
| Nedda | 4.9 | 7.5 | 3.4 | 4.8 | 4.1 | 5.0 |
| Parsifal | 1.9 | 2.0 | 2.1 | 2.1 | 2.1 | 2.1 |
| Simeto | 5.0 | 7.5 | 4.0 | 6.0 | 4.4 | 4.7 |
| Svevo | 8.0 | 8.2 | 6.4 | 7.3 | 5.4 | 5.5 |
| Tresor | 1.5 | 2.3 | 2.5 | 2.8 | 2.8 | 3.2 |
| Varano | 6.2 | 7.9 | 5.6 | 6.4 | 4.9 | 5.3 |
| Green 27 | 5.9 | 7.9 | 6.6 | 7.4 | 4.0 | 4.6 |
| Green 34 | 5.1 | 6.5 | 5.2 | 6.4 | 4.1 | 4.3 |
| Nacori 97 | 4.2 | 6.3 | 3.1 | 4.1 | 3.6 | 4.7 |
| Vitron | 7.4 | 8.8 | 6.7 | 6.7 | 4.8 | 4.8 |
| DHTON 1 | 3.1 | 3.2 | 2.5 | 2.7 | 2.9 | 3.2 |
| Gidara 17a | 5.7 | 7.9 | 6.8 | 7.7 | 4.1 | 4.9 |
| Marjak | 4.0 | 5.0 | 2.8 | 3.5 | 2.5 | 2.4 |
| Arrivato | 1.8 | 1.8 | 1.4 | 1.6 | 2.0 | 2.2 |
| CFR5001 | 2.0 | 2.8 | 2.4 | 2.4 | 2.4 | 2.7 |
| CRDW17 | 2.3 | 1.9 | 1.8 | 2.0 | 3.1 | 3.2 |
| K-39099 | 7.3 | 8.1 | 7.1 | 7.1 | 6.3 | 6.5 |
| Altar-Aos | 3.2 | 3.9 | 2.4 | 2.7 | 2.2 | 2.3 |
| Borli | 6.3 | 7.7 | 6.1 | 7.2 | 4.7 | 4.7 |
| Camacho | 4.5 | 5.3 | 2.3 | 2.7 | 3.1 | 3.2 |
| Gallareta | 1.8 | 1.8 | 2.1 | 2.2 | 1.9 | 2.1 |
| Mexa | 5.0 | 6.9 | 6.0 | 6.7 | 4.2 | 5.3 |
| D940027 | 5.5 | 7.5 | 5.8 | 7.0 | 5.2 | 5.6 |
| D940098 | 3.5 | 4.7 | 4.0 | 4.6 | 3.9 | 4.6 |
| D941038 | 4.6 | 6.0 | 3.7 | 3.7 | 4.6 | 5.0 |
| D95580 | 3.8 | 4.7 | 2.7 | 2.7 | 3.9 | 4.2 |
| Durex | 4.2 | 5.4 | 2.9 | 3.0 | 2.6 | 2.6 |
| Kofa | 7.8 | 8.3 | 6.7 | 6.9 | 5.4 | 5.7 |
| Kronos | 6.3 | 7.7 | 4.5 | 5.7 | 3.2 | 3.2 |
| Langdon Dic 6B | 6.8 | 7.7 | 4.7 | 6.5 | 3.1 | 3.4 |
| Ocotillo | 7.5 | 8.4 | 7.0 | 7.3 | 5.8 | 6.4 |
| Plaza | 4.1 | 3.1 | 2.5 | 2.5 | 5.1 | 5.0 |
| Westbred881 | 8.1 | 8.7 | 7.1 | 7.2 | 6.7 | 6.9 |
| Mean (pop.) | 5.1 | 6.1 | 4.4 | 5.0 | 4.2 | 4.6 |
| Min (pop.) | 1.5 | 1.7 | 1.4 | 1.6 | 1.8 | 2.1 |
| Max (pop.) | 8.4 | 8.9 | 7.7 | 7.7 | 6.9 | 7.3 |
| Average LSD 0.05 | 0.8 | 0.8 | 0.7 | 0.7 | 0.6 | 0.6 |
