# Supplementary material for: Genetic analysis of resistance to stripe rust in durum wheat (Triticum turgidum L. var. durum)
Source: PLoS One. 2018 Sep 19;13(9):e0203283. doi: 10.1371/journal.pone.0203283 (PMC6145575; doi:10.1371/journal.pone.0203283)
Supplement: S7 Table — (DOCX) [file pone.0203283.s010.docx]

# S7 Table Pearson correlations between seedling infection type for the breeding population inoculated with FC, W009 and W015.

|  | FC | W009 | W015 |
| --- | --- | --- | --- |
| FC | 1 |  |  |
| W009 | 0.91 | 1 |  |
| W015 | 0.77 | 0.77 | 1 |
