# Supplementary material for: Genetic analysis of resistance to stripe rust in durum wheat (Triticum turgidum L. var. durum)
Source: PLoS One. 2018 Sep 19;13(9):e0203283. doi: 10.1371/journal.pone.0203283 (PMC6145575; doi:10.1371/journal.pone.0203283)
Supplement: S8 Table — (DOCX) [file pone.0203283.s011.docx]

# S8 Table LSMeans for the final recording and area under the disease progress curve (AUDPC) for all recordings of adult plant resistance for the breeding panel evaluated in Mexico 2013 and 2014.

| Accession | LSMeans 2013 | AUDPC 2013 | LSMeans 2014 | AUDPC 2014 |
| --- | --- | --- | --- | --- |
| Bonaerance Inta Cumenay | 11.7 | 161.7 | 20.0 | 326.7 |
| Bonaerance Quilaco | 50.0 | 583.3 | 43.3 | 589.2 |
| Bonaerance Valverde | 20.0 | 287.5 | 30.0 | 385.0 |
| Buck Ambar | 0.0 | 0.0 | 0.0 | 0.0 |
| Buck Topacio | 5.0 | 81.7 | 8.3 | 128.3 |
| 920334 | 46.7 | 583.3 | 40.0 | 635.8 |
| 940030 | 63.3 | 653.3 | 50.0 | 828.3 |
| 940435 | 26.7 | 284.2 | 40.0 | 595.0 |
| 940955 | 23.3 | 304.2 | 46.7 | 612.5 |
| 950329 | 20.0 | 247.5 | 30.0 | 490.0 |
| 950844 | 13.3 | 174.2 | 30.0 | 437.5 |
| Tamaroi | 10.0 | 136.7 | 13.3 | 186.7 |
| Wollaroi | 8.3 | 99.2 | 20.0 | 262.5 |
| 9661-AF1D | 11.7 | 175.0 | 13.3 | 274.2 |
| 9661-CA5E | 1.7 | 31.7 | 6.7 | 140.0 |
| AC Avonlea | 8.3 | 144.2 | 20.0 | 361.7 |
| AC Melita | 26.7 | 348.3 | 30.0 | 513.3 |
| AC Morse | 46.7 | 648.3 | 40.0 | 665.0 |
| Napoleon | 13.3 | 213.3 | 26.7 | 455.0 |
| AC Navigator | 50.0 | 621.7 | 63.3 | 1015.0 |
| AC Pathfinder | 70.0 | 843.3 | 86.7 | 1260.0 |
| Commander | 33.3 | 410.0 | 40.0 | 700.0 |
| D24-1773 | 13.3 | 187.5 | 26.7 | 431.7 |
| DT513 | 8.3 | 125.0 | 20.0 | 326.7 |
| DT536 | 33.3 | 460.0 | 26.7 | 431.7 |
| CDC Verona | 8.3 | 131.7 | 13.3 | 204.2 |
| DT691 | 33.3 | 435.0 | 43.3 | 711.7 |
| DT695 | 50.0 | 633.3 | 46.7 | 723.3 |
| DT696 | 20.0 | 235.0 | 13.3 | 204.2 |
| DT704 | 30.0 | 423.3 | 30.0 | 583.3 |
| DT705 | 1.7 | 25.0 | 5.0 | 99.2 |
| DT707 | 0.0 | 13.3 | 13.3 | 215.8 |
| DT709 | 43.3 | 533.3 | 30.0 | 560.0 |
| DT710 | 6.7 | 100.0 | 26.7 | 425.8 |
| DT711 | 15.0 | 218.3 | 26.7 | 484.2 |
| Kyle | 15.0 | 231.7 | 16.7 | 239.2 |
| Strongfield | 18.3 | 255.8 | 16.7 | 233.3 |
| Agridur | 76.7 | 853.3 | 66.7 | 1015.0 |
| Ariesol | 13.3 | 200.0 | 8.3 | 128.3 |
| Carioca | 3.3 | 50.0 | 1.7 | 5.8 |
| RABD 93.40 | 11.7 | 175.0 | 20.0 | 373.3 |
| Tetradur | 11.7 | 175.0 | 20.0 | 361.7 |
| Durabon | 3.3 | 56.7 | 8.3 | 151.7 |
| Durafit | 33.3 | 473.3 | 50.0 | 793.3 |
| 44616 | 10.0 | 137.5 | 6.7 | 81.7 |
| 44721 | 6.7 | 87.5 | 13.3 | 169.2 |
| D-73-15 | 1.7 | 25.0 | 5.0 | 105.0 |
| Arcobaleno | 0.0 | 6.7 | 0.0 | 0.0 |
| Bronte | 46.7 | 506.7 | 40.0 | 700.0 |
| Ciccio | 3.3 | 50.0 | 13.3 | 192.5 |
| Colosseo | 20.0 | 229.2 | 13.3 | 221.7 |
| Demetra | 11.7 | 155.8 | 20.0 | 326.7 |
| Duilio | 3.3 | 56.7 | 16.7 | 192.5 |
| Fortore | 8.3 | 118.3 | 23.3 | 268.3 |
| Gianni | 15.0 | 205.8 | 26.7 | 373.3 |
| Grazia | 6.7 | 100.0 | 10.0 | 128.3 |
| Iride | 0.0 | 0.0 | 0.0 | 0.0 |
| Lesina | 10.0 | 150.0 | 20.0 | 303.3 |
| Mongibello | 11.7 | 155.8 | 26.7 | 367.5 |
| Nedda | 13.3 | 167.5 | 20.0 | 256.7 |
| Parsifal | 8.3 | 125.0 | 6.7 | 35.0 |
| Simeto | 1.7 | 25.0 | 16.7 | 204.2 |
| Svevo | 1.7 | 25.0 | 13.3 | 239.2 |
| Tresor | 0.0 | 0.0 | 1.7 | 29.2 |
| Varano | 21.7 | 254.2 | 23.3 | 315.0 |
| Green 27 | 16.7 | 217.5 | 30.0 | 402.5 |
| Green 34 | 5.0 | 55.8 | 16.7 | 262.5 |
| Nacori 97 | 0.0 | 0.0 | 1.7 | 29.2 |
| Vitron | 26.7 | 303.3 | 30.0 | 595.0 |
| DHTON 1 | 3.3 | 50.0 | 5.0 | 46.7 |
| Gidara 17a | 16.7 | 224.2 | 30.0 | 519.2 |
| Marjak | 8.3 | 118.3 | 10.0 | 157.5 |
| Arrivato | 0.0 | 13.3 | 0.0 | 0.0 |
| CFR5001 | 3.3 | 50.0 | 0.0 | 0.0 |
| CRDW17 | 3.3 | 56.7 | 3.3 | 23.3 |
| K-39099 | 43.3 | 560.0 | 60.0 | 892.5 |
| Altar-Aos | 1.7 | 25.0 | 1.7 | 17.5 |
| Borli | 0.0 | 19.2 | 8.3 | 145.8 |
| Camacho | 0.0 | 6.7 | 1.7 | 35.0 |
| Gallareta | 5.0 | 75.0 | 1.7 | 17.5 |
| Mexa | 15.0 | 174.2 | 30.0 | 490.0 |
| D940027 | 23.3 | 330.0 | 26.7 | 490.0 |
| D940098 | 6.7 | 106.7 | 20.0 | 361.7 |
| D941038 | 15.0 | 192.5 | 26.7 | 490.0 |
| D95580 | 3.3 | 56.7 | 10.0 | 186.7 |
| Durex | 13.3 | 180.8 | 10.0 | 134.2 |
| Kofa | 23.3 | 298.3 | 33.3 | 571.7 |
| Kronos | 11.7 | 168.3 | 20.0 | 291.7 |
| Langdon Dic 6B | 30.0 | 360.0 | 50.0 | 740.8 |
| Ocotillo | 43.3 | 540.0 | 53.3 | 956.7 |
| Plaza | 13.3 | 206.7 | 8.3 | 169.2 |
| Westbred881 | 80.0 | 878.3 | 90.0 | 1388.3 |
| Mean (pop.) | 17.6 | 226.9 | 23.1 | 364.0 |
| Min (pop.) | 0.0 | 0.0 | 0.0 | 0.0 |
| Max (pop.) | 80.0 | 878.3 | 90.0 | 1388.3 |
| Average LSD 0.05 | 4.1 | 51.2 | 2.7 | 45.6 |
