# Supplementary material for: Genetic analysis of resistance to stripe rust in durum wheat (Triticum turgidum L. var. durum)
Source: PLoS One. 2018 Sep 19;13(9):e0203283. doi: 10.1371/journal.pone.0203283 (PMC6145575; doi:10.1371/journal.pone.0203283)
Supplement: S9 Table — (DOCX) [file pone.0203283.s012.docx]

# S9 Table Polymorphic SNP markers in the breeding population.

| Chromosome | Length (cM) | Num. of unique map positions ^a^ | Density (Num. of markers/cM) | Max. gap (cM) | Num. of gaps larger than 10 cM |
| --- | --- | --- | --- | --- | --- |
| 1A | 149.0 | 181 | 1.21 | 9.50 | 0 |
| 1B | 176.5 | 252 | 1.43 | 10.80 | 1 |
| 2A | 212.5 | 173 | 0.81 | 12.20 | 2 |
| 2B | 193.6 | 272 | 1.40 | 4.80 | 0 |
| 3A | 184.3 | 169 | 0.92 | 7.10 | 0 |
| 3B | 209.6 | 218 | 1.04 | 10.10 | 1 |
| 4A | 177.3 | 165 | 0.93 | 10.50 | 1 |
| 4B | 135.8 | 193 | 1.42 | 11.10 | 0 |
| 5A | 218.6 | 183 | 0.84 | 6.70 | 0 |
| 5B | 206.2 | 240 | 1.16 | 6.00 | 0 |
| 6A | 131.1 | 184 | 1.40 | 11.10 | 1 |
| 6B | 152.8 | 214 | 1.40 | 5.20 | 0 |
| 7A | 210.6 | 222 | 1.05 | 6.10 | 0 |
| 7B | 212.3 | 226 | 1.06 | 9.90 | 0 |
| Whole genome | 2570.2 | 2892 | 1.13 | 12.20 | 6 |

^a^ 12,237 polymorphic markers were anchored to the consensus map of durum wheat and the number of unique map positions is presented
