# Supplementary material for: Genetic analysis of resistance to stripe rust in durum wheat (Triticum turgidum L. var. durum)
Source: PLoS One. 2018 Sep 19;13(9):e0203283. doi: 10.1371/journal.pone.0203283 (PMC6145575; doi:10.1371/journal.pone.0203283)
Supplement: S10 Table — (DOCX) [file pone.0203283.s013.docx]

S10 Table Summary of significant markers located on chromosome 7B associated with stripe rust resistance in the breeding panel, including seedling reaction to FC, single isolates W009 and W015 and adult plant resistance reaction in Mexico in 2013 and 2014.

| **SNP / SSR ID** | **Chr.** | **Position ^a^ (cM)** | **FC** | | | **W009** | | | **W015** | | | **Mexico (2013)** | | | **Mexico (2014)** | | |
| --- | --- | --- | --- | --- | --- | --- | --- | --- | --- | --- | --- | --- | --- | --- | --- | --- | --- |
|  |  |  | ***P-*value** | **R^2^ (%) ^b^** | **Effect** | ***P-*value** | **R^2^ (%)** | **Effect** | ***P-*value** | **R^2^ (%)** | **Effect** | ***P-*value** | **R^2^ (%)** | **Effect** | ***P-*value** | **R^2^ (%)** | **Effect** |
| *Excalibur_c51720_84* | 7B | 187.5 | 0.0003 | 21.3 | -1.44 | 0.001 | 24.3 | -1.18 | 0.022 | 17.1 | -1.17 | 0.020 | 15.8 | -4.97 | 1.24E-04 | 17.8 | -5.99 |
| *RAC875_c54854_164* | n/a | n/a | 0.0005 | 22.8 | -1.96 | 0.001 | 31.1 | -2.18 | NS |  |  | 0.006 | 17.1 | -5.35 | 2.81E-04 | 16.1 | -6.50 |
| *BS00022162_51* | 7B | 187.5 | 0.0003 | 21.3 | -1.44 | 0.001 | 24.3 | -1.18 | 0.022 | 17.1 | -1.17 | 0.020 | 15.8 | -4.97 | 1.24E-04 | 17.8 | -5.99 |
| *Tdurum_contig61884_836* | 7B | 186 | 0.0001 | 23.1 | 0.71 | NS^c^ |  |  | NS |  |  | NS | | | 2.45E-03 | 11.6 | 5.03 |
| *Tdurum_contig49575_1237* | 7B | 187.5 | 0.0001 | 25.2 | -1.54 | 0.001 | 29.7 | -1.27 | 0.001 | 20.4 | -1.26 | 0.001 | 20.4 | -5.62 | 2.33E-05 | 22.0 | -6.57 |
| *Kukri_c46447_1738* | 7B | 187.5 | 0.0003 | 21.3 | -1.44 | 0.001 | 24.3 | -1.18 | 0.022 | 17.1 | -1.17 | 0.020 | 15.8 | -4.97 | 1.24E-04 | 17.8 | -5.99 |
| *Kukri_c3781_285* | 7B | 187.5 | 0.0001 | 25.2 | -0.77 | 0.001 | 29.7 | -0.64 | 0.001 | 20.4 | -0.63 | 0.001 | 20.4 | -2.81 | 2.33E-05 | 22.0 | -6.57 |
| *BobWhite_c12355_1590* | n/a | n/a | 0.0004 | 23.2 | -1.46 | 0.001 | 24.3 | -1.18 | NS |  |  | 0.038 | 16.2 | -5.06 | 1.03E-04 | 18.2 | -6.01 |
| *Tdurum_contig42586_720* | 7B | 187.5 | 0.0001 | 24.1 | -0.74 | 0.001 | 27.7 | -0.60 | 0.005 | 19.2 | -0.60 | 0.001 | 19.4 | -2.64 | 8.78E-05 | 20.1 | -6.14 |
| *Excalibur_c1070_2327* | 7B | 187.5 | 0.0003 | 21.3 | -1.44 | 0.001 | 24.3 | -1.18 | 0.022 | 17.1 | -1.17 | 0.020 | 15.8 | -4.97 | 1.24E-04 | 17.8 | -5.99 |
| *Tdurum_contig42586_290* | 7B | 187.5 | 0.0001 | 24.1 | -0.74 | 0.001 | 27.7 | -0.60 | 0.005 | 19.2 | -0.60 | 0.001 | 19.4 | -2.64 | 8.78E-05 | 20.1 | -6.14 |
| *Tdurum_contig42586_990* | 7B | 187.5 | 0.0001 | 24.1 | -0.74 | 0.001 | 27.7 | -1.20 | 0.005 | 19.2 | -0.60 | 0.001 | 19.4 | -2.64 | 8.78E-05 | 20.1 | -6.14 |
| *RAC875_rep_c111788_253* | 7B | 186 | 0.0003 | 24.8 | 1.50 | NS |  |  | 0.020 | 17.8 | 1.23 | NS | | | 1.09E-03 | 13.6 | 5.43 |
| *Tdurum_contig49575_1207* | 7B | 187.5 | 0.0004 | 23.2 | -1.46 | 0.001 | 24.3 | -1.18 | NS |  |  | 0.038 | 16.2 | -5.06 | 1.03E-04 | 18.2 | -6.01 |
| *Kukri_c48418_149* | 7B | 187.5 | 0.0001 | 25.5 | -0.66 | 0.001 | 32.3 | -1.10 | 0.011 | 21.1 | -0.20 | 0.005 | 20.5 | -0.50 | 9.82E-05 | 22.6 | -1.22 |
| *cfa2040* | 7B | 184.5 | 0.000003 | 26.9 | -0.92 | NS |  |  | NS |  |  | NS | | | NS |  |  |

^a^ The locations of the markers were determined by the consensus map.

^b^ Percentage of phenotypic variance explained (*r^2^*) and effect of associated alleles.

^c^ Non-significant markers were indicated by not significant (NS).
